# Activin/Nodal signaling and NANOG orchestrate human embryonic stem cell fate decisions by controlling the H3K4me3 chromatin mark

**DOI:** 10.1101/gad.255984.114

**Published:** 2015-04-01

**Authors:** Alessandro Bertero, Pedro Madrigal, Antonella Galli, Nina C. Hubner, Inmaculada Moreno, Deborah Burks, Stephanie Brown, Roger A. Pedersen, Daniel Gaffney, Sasha Mendjan, Siim Pauklin, Ludovic Vallier

**Affiliations:** 1 Wellcome Trust-MRC Stem Cell Institute Anne McLaren Laboratory, Department of Surgery, University of Cambridge, Cambridge CB2 0SZ, United Kingdom;; 2 Wellcome Trust Sanger Institute, Hinxton, Cambridge CB10 1SA, United Kingdom;; 3 Department of Molecular Biology, Radboud University Nijmegen, 6525 GA Nijmegen, The Netherlands;; 4 Laboratory of Molecular Endocrinology, Centro de Investigación Príncipe Felipe, 46012 Valencia, Spain

**Keywords:** Activin/Nodal, H3K4me3, hESCs, SMAD2/3, DPY30, NANOG

## Abstract

Stem cells can self-renew and differentiate into multiple cell types. These characteristics are maintained by the combination of specific signaling pathways and transcription factors that cooperate to establish a unique epigenetic state. Despite the broad interest of these mechanisms, the precise molecular controls by which extracellular signals organize epigenetic marks to confer multipotency remain to be uncovered. Here, we use human embryonic stem cells (hESCs) to show that the Activin–SMAD2/3 signaling pathway cooperates with the core pluripotency factor NANOG to recruit the DPY30-COMPASS histone modifiers onto key developmental genes. Functional studies demonstrate the importance of these interactions for correct histone 3 Lys4 trimethylation and also self-renewal and differentiation. Finally, genetic studies in mice show that Dpy30 is also necessary to maintain pluripotency in the pregastrulation embryo, thereby confirming the existence of similar regulations in vivo during early embryonic development. Our results reveal the mechanisms by which extracellular factors coordinate chromatin status and cell fate decisions in hESCs.

## Abstract (precis)

Bertero et al. used human embryonic stem cells (hESCs) to show that the Activin–SMAD2/3 signaling pathway cooperates with the core pluripotency factor NANOG to recruit the DPY30-COMPASS histone modifiers onto key developmental genes. Functional studies demonstrate the importance of these interactions for correct histone 3 Lys4 trimethylation and also for self-renewal and differentiation. In mice, Dpy30 is also necessary to maintain pluripotency in the pregastrulation embryo.

Stem cells are defined by their ability to propagate indefinitely while retaining the capacity to differentiate into multiple cell types. Such remarkable characteristics are dictated by specific cellular signaling pathways, transcriptional networks, and epigenetic regulators. These elements ultimately direct stem cell fate decisions and thus are essential to generate cells required for not only embryonic development but also adult tissue homeostasis and repair (Blanpain and Fuchs 2009; Young 2011; Clevers 2013). Human pluripotent stem cells (hPSCs) isolated from an embryo at the blastocyst stage (human embryonic stem cells [hESCs]) or obtained from reprogrammed somatic cells (human induced pluripotent stem cells [hIPSCs]) represent an advantageous model to study these regulations at the molecular level. Indeed, hPSCs can grow indefinitely in vitro while preserving their ability to differentiate into the three primary germ layers: neuroectoderm, mesoderm, and endoderm.

The signaling pathways controlling hPSC cell fate are relatively well known, and the TGFβ superfamily has a central function in these mechanisms (Xu et al. 2008; Vallier et al. 2009b,c). In particular, Activin/Nodal signaling and its effector, SMAD2/3, are essential to maintain pluripotency, inhibit neuroectoderm specification, and drive mesendoderm differentiation (Vallier et al. 2005; Brown et al. 2011). Activin–SMAD2/3 can achieve this broad range of functions by interacting with tissue-specific master regulators of cell fate that direct SMAD2/3 to subsets of genomic regulatory domains in a cell-type specific fashion (Mullen et al. 2011). As such, SMAD2/3 cooperates with NANOG in hPSCs to maintain the core pluripotency transcriptional network while interacting with EOMES during mesendoderm differentiation to activate the transcription of master developmental regulators (Vallier et al. 2009a; Teo et al. 2011). However, the mechanism by which SMAD2/3 controls the transcriptional activity of its key target genes in hPSCs remains unknown, and it is especially unclear whether this transcriptional control involves epigenetic mechanisms.

Indeed, hPSCs differ from somatic cells due to a specific epigenetic state characterized by a highly dynamic and accessible chromatin structure, which is lost upon induction of differentiation. This epigenetic landscape includes numerous genes that are simultaneously marked by trimethylation of histone 3 Lys4 (H3K4me3) and H3K27me3, two histone marks that are usually found on active and inactive genes, respectively, and thus are associated with opposite transcriptional activities (Pan et al. 2007). Consequently, “bivalent” H3K4me3–H3K27me3 marks have been proposed to poise developmental genes for rapid activation or silencing upon differentiation (Bernstein et al. 2006). H3K4me3 is deposited by COMPASS complexes, which contain enzymes of the MLL/SETD1 family of histone methyltransferases, while H3K27me3 is imposed by the Polycomb group proteins (Schuettengruber et al. 2007).

Despite the wealth of studies describing the dynamic changes of chromatin histone marks in pluripotent cells and their derivatives, there are several important unanswered questions. First, whether and how extracellular signaling cues control the H3K4me3 and H3K27me3 epigenetic landscape remain to be uncovered. Second, H3K4me3 and H3K27me3 marks could have a direct functional importance in the transcriptional regulation of genes directing cell fate decisions or could be just a consequence of such regulations (Vastenhouw and Schier 2012; Voigt et al. 2013). Finally, studies of H3K4me3 and H3K27me3 function in pluripotency have been performed in mouse ESCs (mESCs), which represent a different pluripotent state than hPSCs (Brons et al. 2007). Overall, the regulation and function of H3K4me3 and H3K27me3 in human pluripotency still need to be elucidated.

Considering the central role of Activin/Nodal signaling in pluripotency, we decided to explore its possible function in the regulation of H3K4me3 and H3K27me3 in hESCs. For that, we performed detailed gene expression profile experiments combined with H3K4me3 and H3K27me3 ChIP-seq (chromatin immunoprecipitation [ChIP] followed by deep sequencing) on hESCs grown in the absence of Activin/Nodal signaling. These experiments showed that inhibition of Activin/Nodal results in a rapid loss of H3K4me3 (but not H3K27me3) on a specific subset of genomic regions associated to master regulators of pluripotency and mesendoderm specification that are subsequently silenced, thus leading to neuroectoderm differentiation. Activin/Nodal signaling achieves this regulation through its effector, SMAD2/3, which cooperates with NANOG to recruit DPY30, a subunit of the COMPASS methyltransferase complexes, on key developmental regulators. Importantly, the relevance of these mechanisms was demonstrated by loss-of-function experiments showing that DPY30 is essential for pluripotency and mesendoderm specification of hESCs. Finally, we demonstrated the importance of our findings in the context of embryonic development by showing that the absence of Dpy30 in the mouse embryo impairs gastrulation of the pluripotent epiblast, thus blocking mesendoderm specification while promoting neuroectoderm differentiation. Taken together, our results identify the fundamental dynamics by which signaling pathways orchestrate transcriptional responses via epigenetic changes to establish the capacity of stem cells to differentiate into specific lineages.

## Results

### Activin/Nodal signaling is necessary to maintain the H3K4me3 mark on a specific subset of genes characterizing the pluripotent state of hESCs

**Figure 1.**
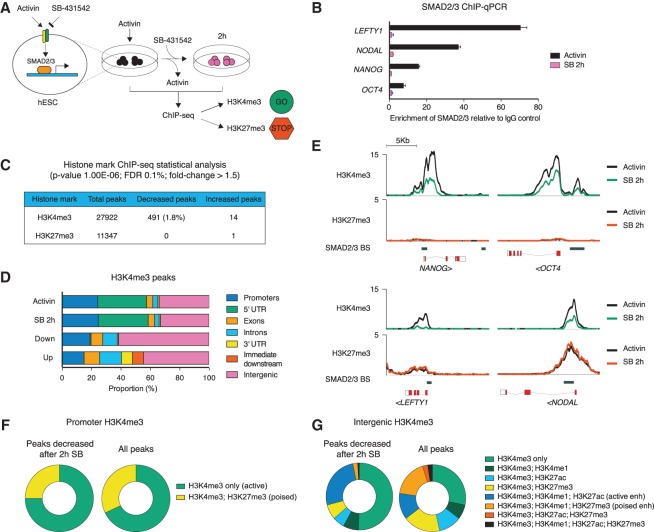
Activin/Nodal regulates H3K4me3 of a subset of genes in hESCs. (*A*) Schematic of the experimental approach. (*B*) ChIP-qPCR for SMAD2/3 on its binding sites associated to the indicated genes (see also *E*) before and after inhibition of Activin/Nodal with SB for 2 h. (*C*) Results of the statistical analysis of ChIP-seq data. (*D*) Annotation of H3K4me3 peaks to genomic features. (*E*) ChIP-seq results for H3K4me3 and H3K27me3 on selected SMAD2/3 target genes before and after inhibition of Activin/Nodal with SB for 2 h. Lines represent read enrichments normalized by million mapped reads and the size of the library. SMAD2/3-binding sites in hESCs are reported (Brown et al. 2011). (*F*) Colocalization of H3K27me3 peaks with H3K4me3 peaks centered in a range of ±5 kb from the closest transcription start site (promoters). (*G*) Colocalization of H3K4me1 and H3K27ac peaks with H3K4me3 peaks centered outside a range of ±5 kb from the closest transcription start site (intergenic).

In order to define the function of Activin/Nodal signaling in the control of the epigenetic status of hESCs, we investigated the effect of SB431542 (SB), an antagonist of the ALK4 and ALK7 type I Activin/Nodal receptors (Fig. 1A), on the deposition of H3K4me3 and H3K27me3 histone marks. Preliminary experiments showed that 2 h of SB treatment of hESCs was the shortest time required to fully block SMAD2/3 phosphorylation and abolish binding to its genomic targets (Fig. 1B; data not shown). Therefore, we performed ChIP-seq for both H3K4me3 and H3K27me3 after 2 h of SB treatment in order to capture the more immediate and hence more likely direct effects of Activin/Nodal inhibition (Fig. 1A; Supplemental Table S1). We found that 491 out of 27,922 H3K4me3 peaks showed a significant decrease after 2 h of SB (fold change ≤ −1.5; Benjamini-Hochberg false discovery rate [FDR] adjusted *P* ≤ 1 × 10^−3^), while only 14 were up-regulated (Fig. 1C; Supplemental Table S1). Importantly, decreased H3K4me3 regions were significantly associated with genes involved in Activin/Nodal signaling and were expressed in the epiblast and endoderm (GREAT analysis) (Supplemental Fig. S1A; Supplemental Table S1). In contrast, we observed almost no significant differences for the 11,347 H3K27me3 peaks identified, with only one region being increased, and none showing a decrease (Fig. 1C). Thus, Activin/Nodal signaling is necessary for maintaining the positive H3K4me3 histone marks on a subset of genes in hESCs, while the deposition of the negative H3K27me3 histone mark appears to be independent of Activin/Nodal.

According to the known typical localization of H3K4me3 (Calo and Wysocka 2013), several of the H3K4me3 peaks that decreased after SB treatment marked proximal promoters and gene transcription start sites (Fig. 1D). These included several known Activin/Nodal target genes such as *NANOG*, *POU5F1/OCT4*, *LEFTY1*, and *NODAL* (Fig. 1E). Interestingly, H3K4me3 was decreased on key pluripotency regulators that are highly expressed and marked by H3K4me3 but not H3K27me3, such as *NANOG*, *POU5F1/OCT4*, *DPPA4*, *GDF3*, and *PRDM14* (Young 2011). However, we observed that inhibition of Activin/Nodal signaling also resulted in impaired H3K4me3 of many genes that only show background expression in hESCs (see Supplemental Fig. S2A for gene expression data) and are marked by both H3K4me3 and H3K27me3, such as *LEFTY1*, *NODAL*, *LEFTY2*, *CER1*, *WNT3*, and *FGF8*. Indeed, 25% of the promoters where H3K4me3 was decreased after 2 h of SB displayed such bivalent marking (Fig. 1F), representing a proportion similar to that of the overall abundance of these elements (32%) (Fig. 1F). Remarkably, the bivalent promoters where H3K4me3 was decreased after SB were associated with genes expressed in the primitive streak, mesoderm, and endoderm (Supplemental Fig. S1B; Supplemental Table S1; Tam and Loebel 2007), thereby supporting the proposed role of bivalent marks in the priming of developmental gene expression (Bernstein et al. 2006). Aside from promoter-associated regions, we observed that ∼60% of H3K4me3 peaks that were down-regulated after 2 h of SB fell outside of gene bodies (Fig. 1D). Inspection of these regions revealed a frequent association with the deposition of H3K4me1 and H3K27ac (Supplemental Fig. S1C), two well-established markers of active enhancers (Calo and Wysocka 2013). Indeed, 25% of intergenic H3K4me3 peaks that decreased after 2 h of SB shared this particular feature (Fig. 1G). In contrast, only 0.02% of them colocalized with H3K4me1 and H3K27me3, a chromatin signature that identifies poised enhancers (Rada-Iglesias et al. 2011), while the overall abundance of such regions was much higher (18%) (Fig. 1G). As such, Activin/Nodal signaling appears to regulate H3K4me3 specifically on active distal enhancers but not on poised ones.

Importantly, inhibition of Activin/Nodal signaling for 2 h specifically impaired H3K4me3 but not H3K4me2 or H3K4me1 levels on both promoter and enhancer regions (Supplemental Fig. S1D). Indeed, H3K4me2 and H3K4me1 levels were either unchanged or increased on most of the regions that we analyzed, with the only exception being *LEFTY1*, where both H3K4me3 and H3K4me2 levels were decreased. Moreover, histone 3 enrichment was unaffected by inhibition of Activin/Nodal signaling (Supplemental Fig. S1D), demonstrating that the observed reduction of H3K4me3 levels was not due to nucleosome repositioning. Overall, these results suggest that inhibition of Activin/Nodal results in specific H3K4me3 demethylation.

Interestingly, 21 of the 491 H3K4me3 peaks that decreased upon Activin/Nodal signaling inhibition directly overlapped with SMAD2/3-binding sites (*P* < 1 × 10^−4^ as measured by genomic association test [GAT]). Among others, canonical SMAD2/3 target genes such as *NANOG*, *POU5F1/OCT4*, *NODAL*, and *LEFTY1* showed this association (Fig. 1E), and, indeed, a decrease of H3K4me3 after 2 h of SB on such regions correlated with loss of SMAD2/3 binding (Fig. 1B). Moreover, regions with decreased H3K4me3 after Activin/Nodal inhibition were significantly associated with nearby SMAD2/3-binding sites (27% and 100% were, respectively, 10 kb or 100 kb upstream of/downstream from the closest SMAD2/3-binding site; GAT, *P* < 1 × 10^−4^ and *P* < 0.033, respectively). This observation is in agreement with previous reports that showed how SMAD2/3 regulates the expression of its target genes mostly by binding to distal enhancers rather than proximal promoters (Brown et al. 2011; Kim et al. 2011).

Taken together, these data suggest that Activin/Nodal signaling could control the expression of master regulators of both pluripotency and germ layer specification by maintaining H3K4me3 on both gene promoters and intergenic enhancers.

### Activin/Nodal signaling maintains H3K4me3 histone marks that are functionally important for pluripotency and cell fate decisions

**Figure 2.**
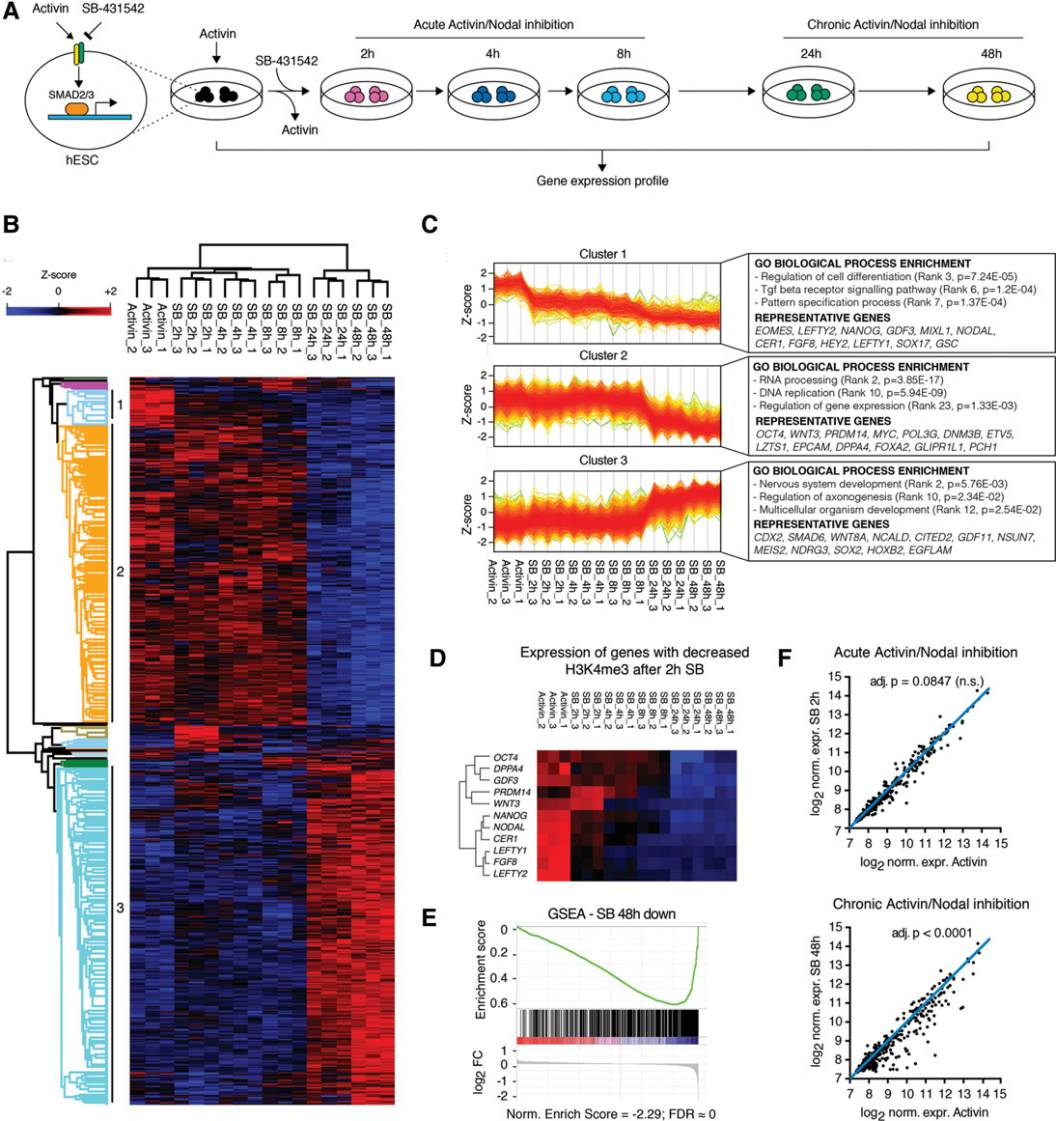
Dynamics of the transcriptional response to Activin/Nodal inhibition and their relationship with epigenetic changes. (*A*) Schematics of the experimental approach. (*B*) Euclidean hierarchical clustering of differentially expressed microarray probes across a time course of Activin/Nodal inhibition in hESCs (top 10% ranked by Hotelling *T*^2^ statistic). *Z*-scores indicate the differential expression measured in number of standard deviations from the average level across all the time points. The three major probe clusters are indicated. (*C*) Expression profiles of probes in the clusters indicated in *B*. Selected results of gene enrichment analysis and representative genes for each cluster are reported (Supplemental Table S2 contains the complete set of results). (*D*) As in *B*, but only selected representative genes that showed decreased H3K4me3 upon 2 h of inhibition of Activin/Nodal signaling are reported. (*E*) Gene set enrichment analysis (GSEA) for genes whose expression was decreased after 48 h of SB in the list of H3K4me3-associated genes ranked by the differential H3K4me3 enrichment before or after 2 h of SB. (*F*) Expression of the genes closest to the H3K4me3 peaks decreased after 2 h of SB. The blue lines indicate no expression change. The significance of expression differences after 2 h or 48 h of SB treatment versus Activin as calculated by Dunn's multiple comparisons tests is shown (see Supplemental Fig. S2B for results for other time points).

To test the functional relevance of H3K4me3 loss after SB treatment, we investigated the transcriptional dynamics resulting from both acute and chronic Activin/Nodal signaling inhibition. Accordingly, we performed gene expression microarrays of hESCs grown in the presence of Activin or SB for 2 h, 4 h, 8 h, 24 h, and 48 h (Fig. 2A; Supplemental Table S2). Hierarchical clustering of differentially expressed probes across the time course (top 10% of probes ranked by their Hotelling *T*^2^ statistic from the timecourse R package) identified three main clusters, two of them containing genes whose expression was decreased after inhibition of Activin/Nodal (Fig. 2B,C). As expected, these clusters were significantly enriched in genes associated with TGFβ signaling, regulation of cell differentiation, and cell cycle (gene enrichment analysis) (Fig. 2C; Supplemental Table S2). However, these two clusters differed in both their relative size and the dynamics of the transcriptional inhibition: The first smaller cluster started to decrease in expression already after 2 h, while the second bigger cluster was significantly affected only after 24 h. Importantly, both clusters presented significant overlap with genes bound by SMAD2/3 (70 out of 233 for cluster 1 and 444 out of 1819 for cluster 2, hypergeometric test *P* = 1.88 × 10^−11^ and *P* = 7.09 × 10^−40^, respectively; SMAD2/3-bound genes from Brown et al. 2011) and contained several well-known SMAD2/3 direct targets such as *LEFTY1*, *NODAL*, *NANOG*, *SOX17*, *EOMES*, and *GSC* (cluster 1) and *POU5F1/OCT4*, *DPPA4*, and *EPCAM* (cluster 2). Importantly, these two clusters included not only several pluripotency factors but also regulators of mesendoderm differentiation (like *LEFTY1*, *NODAL*, *SOX17*, *EOMES*, and *GSC*) that are expressed at background levels in hESCs (Supplemental Fig. S2A reports the Ct values for such genes), in agreement with the known “primed” pluripotency status of hESCs (Nichols and Smith 2009). As such, Activin/Nodal inhibition not only reduces expression of pluripotency genes but also abolishes the primed expression of mesendoderm regulators. Conversely, a third major cluster represented transcripts induced after 24–48 h of Activin/Nodal inhibition (Fig. 2B,C). This was significantly enriched in genes involved in neural development and contained several known SMAD2/3-inhibited factors such as *CDX2*, *WNT8A*, *EGFLAM*, *MEIS2*, and *CITED2* (Fig. 2C; Supplemental Table S2). Importantly, quantitative PCR (qPCR) experiments on a subset of genes from each of these three clusters validated the accuracy of the microarray analyses (Supplemental Fig. S2A). Overall, these observations showed that inhibition of Activin/Nodal signaling leads to both rapid and delayed transcriptional responses that regulate expression of genes involved in pluripotency and cell fate decisions.

We next investigated the relationship between H3K4me3 mark reduction and the transcriptional effects induced by Activin/Nodal inhibition. Interestingly, 108 out of 415 genes associated to decreased H3K4me3 after 2 h of SB were among the top 10% of differentially expressed genes across the kinetics of Activin/Nodal inhibition (Supplemental Table S2). These included several genes crucially required for pluripotency (such as *NANOG*, *POU5F1/OCT4*, *DPPA4*, *GDF3*, and *PRDM14*) as well as regulators of mesendoderm differentiation (such as *LEFTY1*, *LEFTY2*, *NODAL*, *CER1*, *WNT3*, and *FGF8*). These genes belonged to either cluster 1 or cluster 2 described above and were transcriptionally repressed upon inhibition of Activin/Nodal signaling (Fig. 2D), in agreement with the known role of H3K4me3 as a histone mark that promotes gene expression (Ruthenburg et al. 2007). Gene set enrichment analysis (GSEA) confirmed that genes down-regulated after inhibition of Activin/Nodal for 48 h were significantly associated with regions in which H3K4me3 was reduced after 2 h of SB (Fig. 2E). Of note, a transcriptional decrease in genes showing reduced H3K4me3 after 2 h of SB occurred progressively and reached the highest significance at 48 h (Fig. 2F; Supplemental Fig. S2B), suggesting that changes in the H3K4me3 mark preceded the decrease in gene expression. Thus, Activin/Nodal signaling could maintain the expression of key developmental regulators by controlling the deposition of H3K4me3 on the corresponding genomic regions.

In order to confirm and extend this observation, we monitored the levels of H3K4me3 and H3K27me3 by ChIP-qPCR after 2 h, 4 h, and 8 h of SB treatment on a panel of genes transcriptionally down-regulated or up-regulated after inhibition of Activin/Nodal. Strikingly, H3K4me3 was impaired already after 2 h of SB on both quickly (cluster 1) or slowly (cluster 2) transcriptionally down-regulated genes, while up-regulated ones (cluster 3) were not affected (Supplemental Fig. S2C). Interestingly, none of the genes analyzed showed any significant change in the levels of H3K27me3 (Supplemental Fig. S2D). These observations confirmed that loss of H3K4me3 associated with SMAD2/3 disappearance precedes transcriptional impairment on a subset of genomics targets. This suggests a functional importance of H3K4me3 histone mark regulation downstream from Activin/Nodal signaling to maintain the expression of a core network of genes controlling pluripotency and differentiation potency.

### SMAD2/3 interacts with DPY30 to maintain H3K4me3 marks on pluripotency and mesendoderm genes

**Figure 3.**
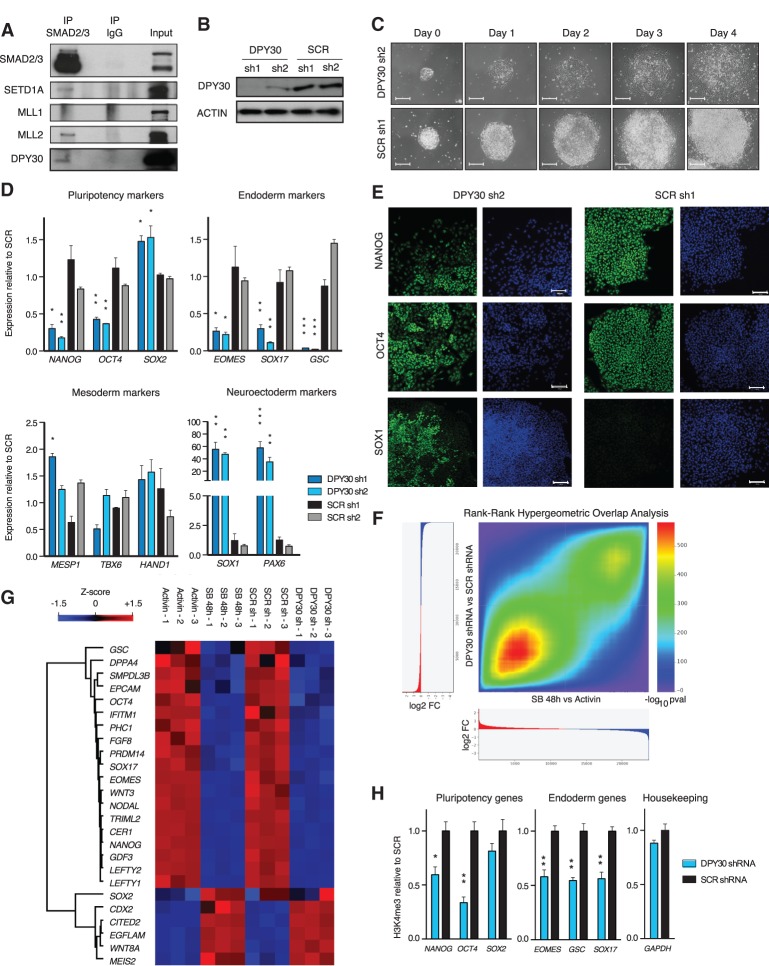
DPY30 is required for H3K4me3 and expression of SMAD2/3 target genes. (*A*) Western blots of SMAD2/3 or control (IgG) immunoprecipitations (IP) from nuclear extracts of hESCs. Input is 5% of the material used for immunoprecipitation. (*B*) Western blots in stable DPY30 knockdown (KD) hESC lines or controls (cells expressing a scramble [SCR] shRNA). (*C*) Phase-contrast images of the same DPY30 knockdown or control hESC colonies after the indicated number of days from the cell split (day 0). Bars, 200 μm. (*D*) Gene expression qPCR in DPY30 knockdown or control hESCs. Note that *SOX2* is both a pluripotency and a neuroectoderm marker. For each gene, significant differences versus both SCR sh1 and SCR sh2 (only the highest *P*-value is shown) as calculated by one-way ANOVA are reported. (*E*) Immunofluorescences for the indicated proteins (green) or nuclear staining (DAPI, blue) in DPY30 knockdown or control hESCs. Bars, 100 μm. (*F*) Rank–rank hypergeometric overlap analysis (RRHO) for genes ranked by their differential expression after DPY30 knockdown or inhibition of Activin/Nodal for 48 h with SB. Color-coded log_10_ *P*-values indicate the significance of the overlap between genes in the two conditions, considering hypergeometric tests. (*G*) Heat map showing changes in gene expression of selected SMAD2/3 target genes after DPY30 knockdown or 48 h of SB. *Z*-scores were separately calculated for each experiment. (*H*) ChIP-qPCR for H3K4me3 on SMAD2/3 target genes in hESCs transiently transfected for 48 h with a DPY30 shRNA or a control shRNA. For each gene, significant differences versus SCR as calculated by *t*-test are reported.

Having defined the importance of Activin/Nodal signaling in maintaining H3K4me3 on key regulatory genes, we sought to understand the underlying molecular mechanism. The association between decreased H3K4me3 and SMAD2/3-binding sites suggested that Activin/Nodal signaling effectors could be directly involved in the deposition of H3K4me3. Thus, we decided to test whether SMAD2/3 could interact with the COMPASS complexes, which are responsible for the deposition of this histone modification. The COMPASS H3K4 methyltransferases belong to the MLL/SETD1 family and form six functional complexes classified into three subgroups, all of which include common WRAD (WDR5, RBBP5, ASHL2, and DPY30) cofactors (Ruthenburg et al. 2007; Ernst and Vakoc 2012). Among the enzymes, SETD1A, MLL1/KMT2A, and MLL2/KMT2B have the highest expression in hESCs (data not shown), and coimmunoprecipitation experiments revealed that SMAD2/3 bound to SETD1A and MLL2/KMT2B, but not MLL1/KMT2A, in hESCs (Fig. 3A).

In order to evaluate the functional relevance of such interactions in the context of Activin/Nodal-dependent H3K4me3 marks and gene expression, we decided to knock down the expression of the common COMPASS cofactor DPY30. Indeed, DPY30 is required for efficient COMPASS-dependent H3K4me3 but not H3K4 mono- and dimethylation, in contrast with the other members of the WRAD module, which are also important for H3K4me2 and H3K4me1 deposition (Ernst and Vakoc 2012). Moreover, since it is a member of all of the COMPASS complexes, impairment of DPY30 should prevent compensatory mechanisms between catalytic subunits as previously reported (Jiang et al. 2011). As such, knockdown of DPY30 has been used as a powerful genetic tool to specifically impair H3K4me3 deposition in different cell types (Jiang et al. 2011; Yang et al. 2014), since specific inhibitors of this histone modification are not currently available. hESCs were stably transfected with vectors expressing shRNAs directed against DPY30 (Supplemental Fig. S3A), and individual sublines showing impaired levels of DPY30 at the RNA and protein levels were isolated and expanded for further analyses (Fig. 3B; Supplemental Fig. S3B). Of note, all of the results described below were confirmed with two separate shRNAs to exclude off-target effects. Interestingly, we obtained a reduced number of sublines after transfection of DPY30 shRNAs when compared with hESCs transfected with a vector expressing a scramble (SCR) shRNA (Supplemental Fig. S3C). This suggested that the absence of DPY30 expression might not be compatible with the self-renewal of hESCs. Accordingly, expansion of hESC subline knockdown for DPY30 (DPY30 knockdown hESCs) was challenging due to a markedly increased background of differentiation and a slower proliferation rate (Fig. 3C; Supplemental Fig. S3D). Interestingly, apoptosis was not increased in these cells, suggesting that DPY30 was not required for hESC survival (Supplemental Fig. S3E). More importantly, DPY30 knockdown hESCs displayed a reduced alkaline phosphatase activity (Supplemental Fig. S3F) and a reduced expression of pluripotency and endoderm markers, with a parallel increase in neuroectoderm genes (Fig. 3D). These results were confirmed at the protein level by Western blot, flow cytometry, and immunostaining (Fig. 3E; Supplemental Fig. S3G,H). Therefore, these observations indicated that DPY30 is necessary to preserve the pluripotent state of hESCs by maintaining the expression of pluripotency markers and blocking the expression of neuroectoderm genes.

Interestingly, the phenotype of DPY30 knockdown hESCs closely resembled the effect of Activin/Nodal signaling inhibition (Smith et al. 2008). This similarity was confirmed at a global level by microarray analyses showing that genes down-regulated after DPY30 knockdown were significantly associated with TGFβ signaling and significantly overrepresented for genes required for embryonic development, while up-regulated transcripts were overrepresented for those involved in craniofacial and neural development (gene enrichment analysis) (Supplemental Table S3). Moreover, the transcriptional profile of DPY30 knockdown hESCs had a highly significant overlap with the one resulting from both 2 h (data not shown) and 48 h (Fig. 3F) of SB treatment (highest hypergeometric test *P* < 1 × 10^−500^, as calculated using rank–rank hypergeometric overlap [RRHO] analysis). Indeed, all of the genes that we validated by qPCR to be increased or decreased during SB treatment (Supplemental Fig. S2A) followed the same trend in DPY30 knockdown cells, including well-known SMAD2/3 targets such as *LEFTY1*, *NODAL*, *NANOG*, *POU5F1/OCT4*, *CDX2*, and *WNT8A* (Fig. 3G; Supplemental Table S3). Together, these data suggested a direct functional interaction between DPY30 and the Activin/Nodal–SMAD2/3 signaling pathway.

We then evaluated whether the transcriptional effects associated with diminished DPY30 expression correlated with changes in H3K4me3 levels. ChIP-qPCR on both pluripotency and endoderm regulators activated by SMAD2/3 and down-regulated after DPY30 knockdown showed reduced levels of H3K4me3 (Supplemental Fig. S3I). Interestingly, DPY30 knockdown did not affect H3K4me3 on a diversity of loci, including housekeeping genes such as *GAPDH* (Supplemental Fig. S3I), thereby suggesting that the requirement for high levels of DPY30 in hESCs is gene-specific. Transient transfection for 48 h of a DPY30 shRNA also resulted in a similar gene-specific reduction of H3K4me3 on SMAD2/3 target genes (Fig. 3H), indicating that histone marks quickly decreased after DPY30 knockdown. This event preceded changes in gene expression (Supplemental Fig. S3J), further supporting the notion that this epigenetic change induced transcriptional impairment. Collectively, these findings suggest that DPY30 and SMAD2/3 cooperate to preserve the H3K4me3 histone mark on several crucial Activin/Nodal signaling target genes and that this mechanism is necessary to maintain pluripotency of hESCs.

### Mesendoderm differentiation potency of hESCs relies on H3K4me3 deposition

**Figure 4.**
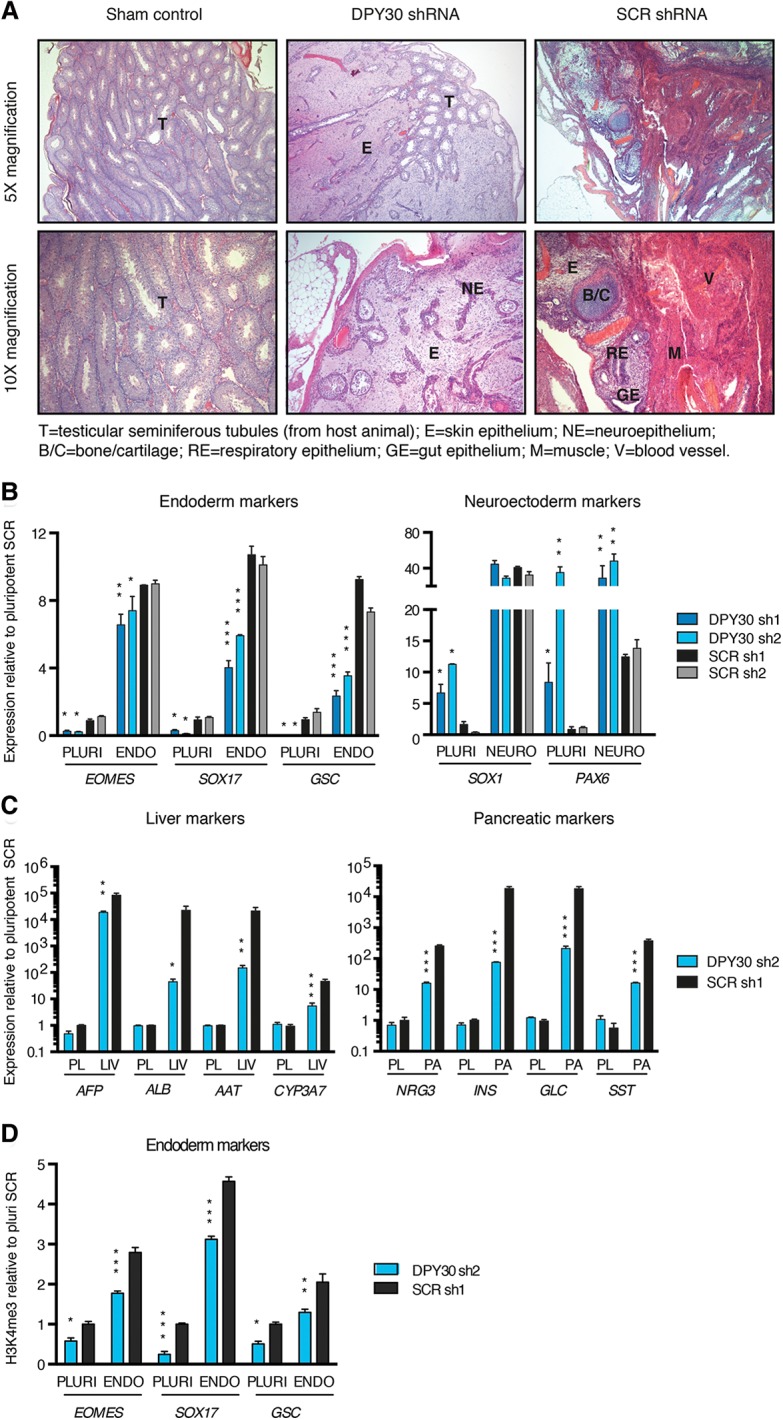
DPY30 is required for mesendoderm differentiation of hESCs. (*A*) Hematoxylin and eosin histological staining of normal testis tissue (sham control) or teratomas derived from DPY30 knockdown (KD) or control hESCs. (*B*) Gene expression qPCR in DPY30 knockdown or control hESCs (cells expressing a SCR shRNA) before (PLURI) or after in vitro directed differentiation toward endoderm or neuroectoderm. For each gene, significant differences versus both SCR sh1 and SCR sh2 in the same condition (only the highest *P*-value is shown) as calculated by two-way ANOVA are reported. (*C*) Gene expression qPCR as in *A* but either before (pluripotent [PL]) or after liver (LIV) or pancreas (PA) differentiation. For each gene, significant differences versus SCR in the same condition as calculated by two-way ANOVA are reported. (*D*) ChIP-qPCR for H3K4me3 in DPY30 knockdown or control hESCs before (PLURI) or after endoderm differentiation. Significant differences versus SCR sh1 in the same condition as calculated by two-way ANOVA are reported.

Considering the effect of DPY30 knockdown in undifferentiated hESCs, we decided to assess the capacity of differentiation of DPY30 knockdown hESCs by performing in vivo teratoma assays (Supplemental Fig. S4A). Strikingly, teratomas derived from DPY30 knockdown hESCs were small and failed to completely invade the testicular capsule of immuno-deficient mice, in contrast to those grown from SCR shRNA control hESCs (Fig. 4A; Supplemental Fig. S4B). Moreover, the resulting tissue was composed of only neuroectodermal lineages, such as epithelial and neuroepithelial cells, but lacked mesoderm and endoderm derivatives (Fig. 4A; Supplemental Fig. S4B). These results showed that a decrease in DPY30 expression limited the capacity of hESCs to both self-renew and differentiate into all derivatives of the three germ layers.

To precisely characterize this differentiation potency defect, DPY30 knockdown hESCs were grown in chemically defined culture conditions directing differentiation toward endoderm and neuroectoderm (Supplemental Fig. S4C; Vallier et al. 2009b). qPCR analyses for the expression of lineage-specific markers proved that DPY30 knockdown hESCs responded poorly to endoderm differentiation, while neuroectoderm specification was more efficient (Fig. 4B; Supplemental Fig. S4D). Importantly, reduced mRNA levels for endoderm genes correlated with impaired RNA polymerase II and Mediator recruitment on their transcription start sites (Supplemental Fig. S4E) and with reduced levels of the elongation marker H3K36me3 on their gene bodies (Supplemental Fig. S4E), thereby suggesting that DPY30 knockdown caused transcriptional impairment. Moreover, SMAD2/3 binding to endoderm genes was also reduced in DPY30 knockdown hESCs differentiated into endoderm (Supplemental Fig. S4E). Impaired endoderm specification was confirmed at the protein level by Western blot and flow cytometry (Supplemental Fig. S4F,G) and resulted in the inability of DPY30 knockdown hESCs to further differentiate into mature endoderm-derived lineages such as the liver and pancreas (Fig. 4C). Finally, the impaired expression of endoderm markers in DPY30 knockdown hESCs was associated with decreased H3K4me3 (Fig. 4D), thereby confirming that transcriptional inhibition provoked by the decrease in DPY30 expression is associated with epigenetic deregulation.

Overall, these results suggested that loss of H3K4me3 on developmental genes in hESCs impairs their capacity to differentiate into mesendoderm and thus demonstrate the functional importance of the correct deposition of this epigenetic mark for the induction of differentiation markers and early cell fate decisions.

### SMAD2/3 cooperates with NANOG to recruit H3K4 methyltransferases on Activin/Nodal*-*responsive genes

**Figure 5.**
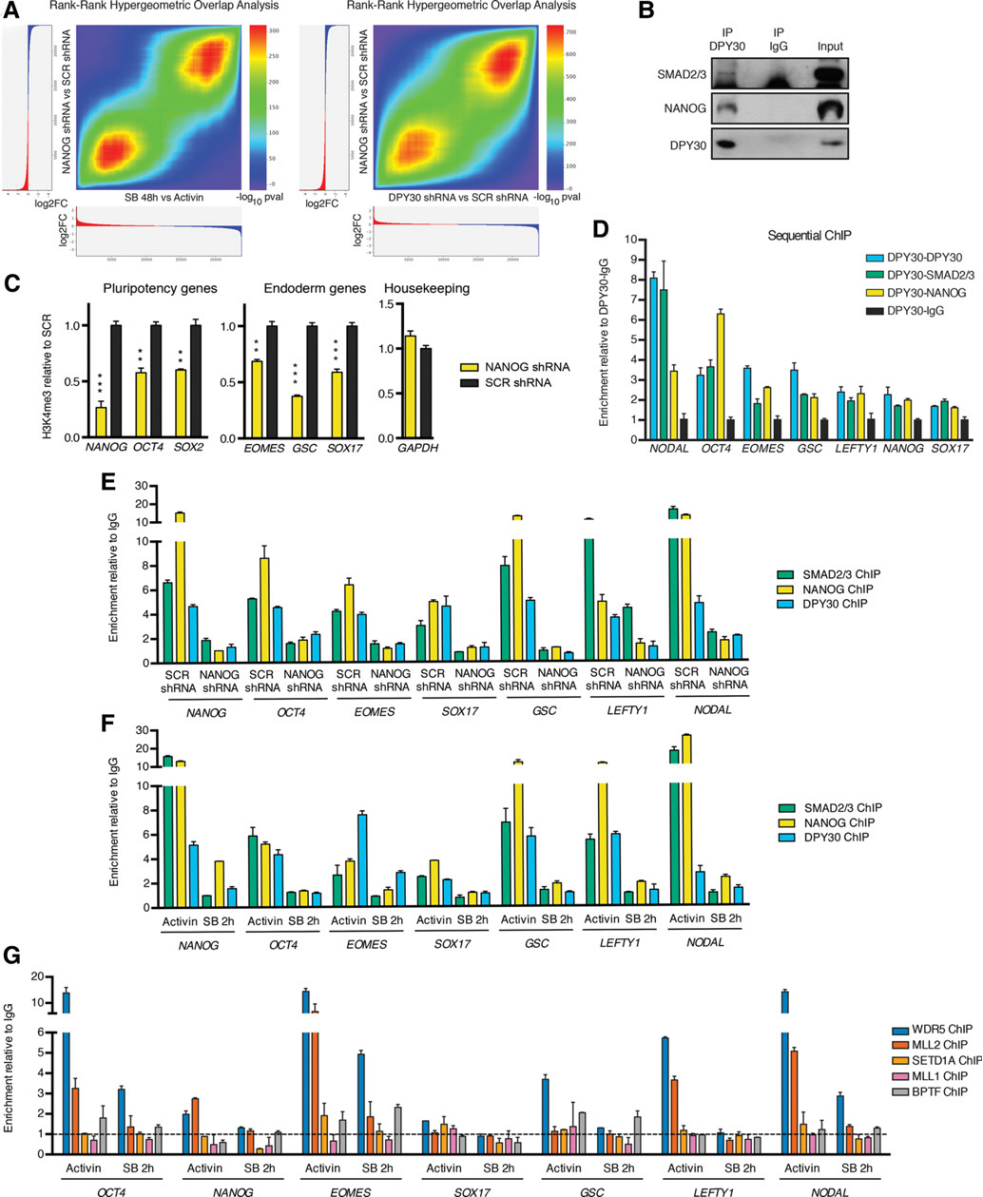
SMAD2/3 and NANOG recruit DPY30 onto their genomic targets. (*A*) RRHO analysis for genes ranked by their differential expression after NANOG knockdown (KD), DPY30 knockdown, or inhibition of Activin/Nodal for 48 h with SB. (*B*) Western blots of DPY30 or control (IgG) immunoprecipitations (IP) from nuclear extracts of hESCs. Input is 5% of the material used for immunoprecipitation. (*C*) ChIP-qPCR for H3K4me3 in NANOG knockdown or control hESCs. For each gene, significant differences versus SCR as calculated by *t*-test are reported. (*D*) Sequential ChIP-qPCR for DPY30 followed by SMAD2/3, NANOG, or control (IgG) ChIP. (*E*) ChIP-qPCR for the indicated proteins in NANOG knockdown or control hESCs. (*F*,*G*) ChIP-qPCR for the indicated proteins in hESCs before and after inhibition of Activin/Nodal with SB for 2 h. ChIP-qPCR results in *D*–*G* are representative of three independent experiments, and the location of primers used is shown in Supplemental Figure S5G.

The decrease in DPY30 expression in hESCs not only recapitulated the effects of Activin/Nodal inhibition but also closely mimicked the consequences of NANOG knockdown in hESCs (Vallier et al. 2009a). Indeed, NANOG knockdown impaired the expression of pluripotency markers and mesendoderm markers while inducing neuroectoderm genes (Supplemental Fig. S5A). Furthermore, microarray analysis demonstrated a significant similarity between the transcriptional responses to NANOG knockdown, the inhibition of Activin/Nodal signaling for 48 h, and DPY30 knockdown (highest hypergeometric *P* < 1 × 10^−300^ and 1 × 10^−700^, respectively) (Fig. 5A; Supplemental Table S4; Supplemental Fig. S5B). While these results were in agreement with the known function of NANOG as a SMAD2/3 cofactor (Mullen et al. 2011; Teo et al. 2011), they also suggested a previously unknown overlap between the SMAD2/3–DPY30 and NANOG-dependent transcriptional networks. Supporting this notion, coimmunoprecipitation experiments showed that NANOG could be found in protein complexes containing DPY30 and SMAD2/3 and that DPY30 and SMAD2/3 coimmunoprecipitated with NANOG (Fig. 5B; Supplemental Fig. S5C). Moreover, NANOG knockdown hESCs displayed lower levels of H3K4me3 on pluripotency and endoderm genes bound by NANOG and SMAD2/3 (Fig. 5C), further suggesting a functional link with SMAD2/3–DPY30. Considered together, these results suggest the existence of a complex between SMAD2/3, NANOG, and DPY30, which we refer to here as S/N/D.

To further validate this hypothesis, we performed sequential ChIP experiments that demonstrated that DPY30 cobinds with both SMAD2/3 and NANOG onto chromatin regions associated to key regulators of hESC pluripotency and differentiation (Fig. 5D). We then investigated whether genomic binding of DPY30 and SMAD2/3 requires the presence of NANOG in the complex. Strikingly, NANOG knockdown impaired binding of both DPY30 and SMAD2/3 on pluripotency and endoderm genes (Fig. 5E), suggesting that NANOG could recruit DPY30 and SMAD2/3 onto its genomic targets. On the other hand, inhibition of SMAD2/3 binding with 2 h of SB treatment also resulted in loss of both NANOG and DPY30 binding (Fig. 5F), indicating that SMAD2/3 was also necessary for the recruitment of both NANOG and DPY30. Finally, we evaluated the formation of the S/N/D complex by coimmunoprecipitation in the presence or absence of Activin/Nodal signaling (Supplemental Fig. S5D) or of NANOG expression (Supplemental Fig. S5E). In both cases, the interaction with DPY30 was impaired, suggesting that SMAD2/3 phosphorylation and the expression of NANOG facilitate the assembly of the S/N/D complex. Taken together, these results implied that SMAD2/3 and NANOG depend on each other to efficiently bind Activin/Nodal-responsive genes and recruit DPY30 on genes characterizing the pluripotent state of hESCs.

We then investigated whether S/N/D binding correlated with histone methyltransferase recruitment. First, we confirmed that COMPASS complexes are recruited in an Activin/Nodal-dependent manner onto chromatin regions bound by S/N/D, as measured by ChIP for WDR5 before and after inhibition of Activin/Nodal signaling for 2 h (Fig. 5G). Furthermore, we observed that the catalytic subunit MLL2/KMT2B, but not SETD1A or MLL1/KMT2A, bound to most S/N/D target loci in an Activin/Nodal-dependent manner (Fig. 5G). Accordingly, WDR5 and MLL2/KMT2B appeared to be part of the S/N/D complex, as measured by coimmunoprecipitation (Supplemental Fig. S5C). Interestingly, transient knockdown of DPY30 did not result in impaired recruitment of WDR5 or MLL2/KMT2B onto S/N/D target regions (Supplemental Fig. S5F). This observation suggests that the effects of DPY30 knockdown are the results of impaired H3K4me3 activity of COMPASS complexes rather than their impaired recruitment onto the chromatin, in agreement with what was reported in previous studies (Jiang et al. 2011; Ernst and Vakoc 2012; Yang et al. 2014). Finally, it was recently reported that DPY30 can also be found in the NURF nucleosome remodeling complex (van Nuland et al. 2013), and thus we decided to investigate the binding profile of the NURF catalytic subunit BPTF on Activin/Nodal target genes. In contrast to the COMPASS complexes, BPTF enrichment on S/N/D-bound regions was limited and independent from the presence of Activin/Nodal signaling (Fig. 5G), arguing against a role for the NURF complex in the regulation of S/N/D targets. Overall, these experiments support a model in which SMAD2/3 and NANOG binding onto Activin/Nodal target genes induces recruitment of histone methyltransferases that regulate H3K4me3 deposition (Supplemental Fig. S5G).

### DPY30, NANOG, and SMAD2/3 cooperation is necessary for H3K4me3 deposition on a core transcriptional network that characterizes pluripotency

**Figure 6.**
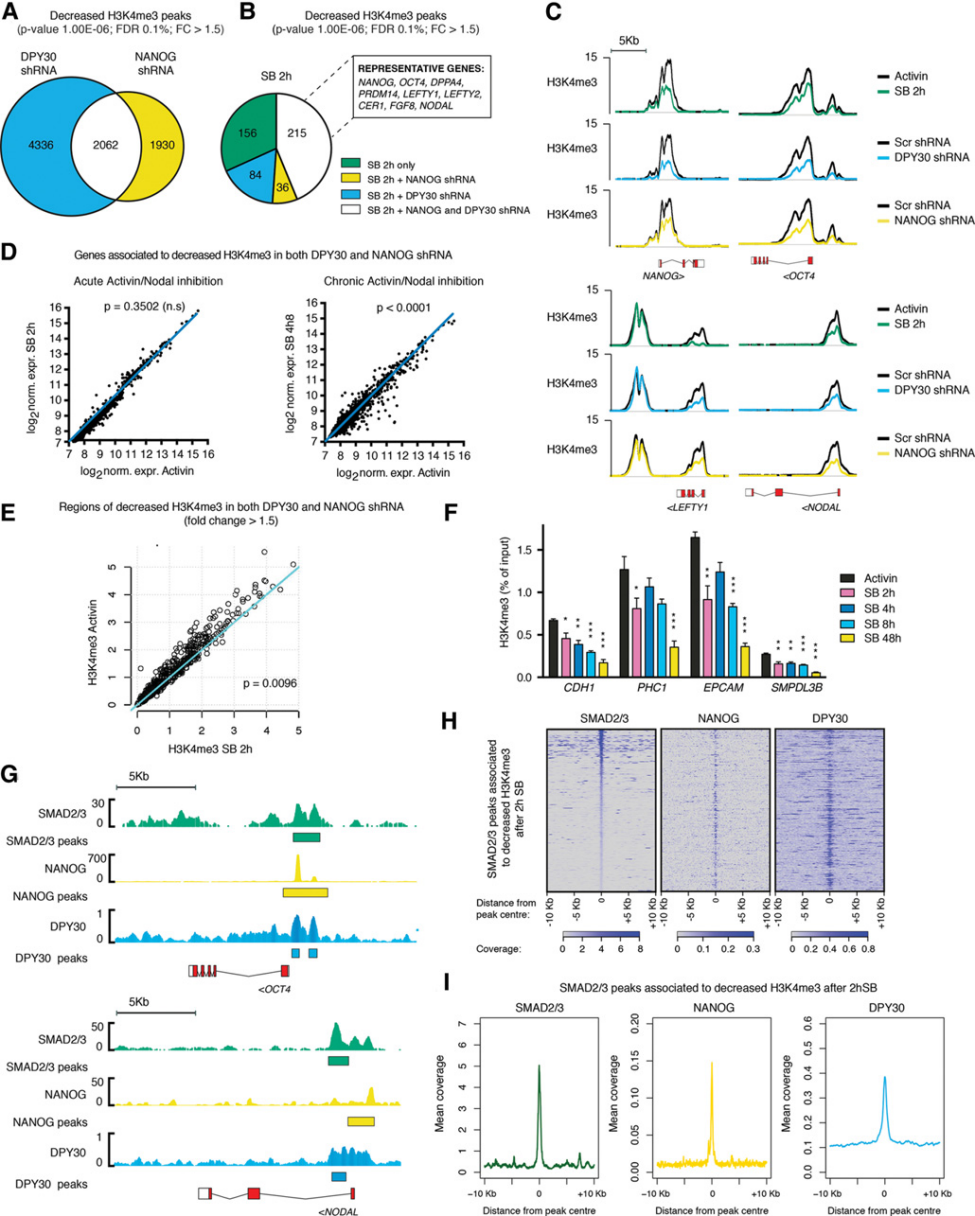
SMAD2/3, NANOG, and DPY30 control H3K4me3 on a subset of shared genes. (*A*) Overlap between H3K4me3 peaks significantly down-regulated after DPY30 knockdown (KD) and NANOG knockdown. (*B*) The proportion of H3K4me3 peaks significantly down-regulated after 2 h of Activin/Nodal inhibition with SB that were also similarly affected by DPY30 knockdown, NANOG knockdown, or both treatments. Representative genes associated to peaks down-regulated in all conditions are reported. (*C*) Examples of ChIP-seq results for H3K4me3 on selected SMAD2/3 target genes before or after DPY30 knockdown, NANOG knockdown, or SB treatment for 2 h. Lines represent read enrichment normalized by million mapped reads and the size of the library. (*D*) Expression of the genes closest to H3K4me3 peaks significantly down-regulated by >50% after both DPY30 knockdown and NANOG knockdown but not decreased to the same extent after 2 h of SB. The significance of expression differences versus Activin as calculated by Welch's *t*-test is reported. (*E*) Average normalized H3K4me3 read enrichment in three biological replicates before or after 2 h of SB. Data refer only to H3K4me3 peaks described in *D*. The level of significant change versus Activin as calculated using Welch's *t*-test is reported. (*F*) ChIP-qPCR for H3K4me3 before and after inhibition SB for 2, 4, 8, or 48 h. The genes analyzed belong to the group described in *D*. For each gene, significant changes versus Activin as calculated by one-way ANOVA are reported. (*G*) Examples of ChIP-seq coverage (*top*) and peaks (*bottom*) for SMAD2/3, NANOG, and DPY30 in hESCs. (*H*) Heat maps of coverage for SMAD2/3, NANOG, and DPY30 ChIP-seq relative to the SMAD2/3 peaks located 100 kb upstream of/downstream from H3K4me3 regions decreased after 2 h of SB (530 peaks). (*I*) Mean coverage plots for the peaks considered in *H*.

Having established a model for the functional interactions between SMAD2/3, NANOG, and DPY30 on a subset of Activin/Nodal-responsive genes, we decided to extend our analysis to a genome-wide scale by performing H3K4me3 ChIP-seq in DPY30 knockdown and NANOG knockdown hESCs (Supplemental Fig. S6A; Supplemental Table S5). While both conditions induced a large number of significant differences, deregulation of H3K4me3 was still specific to selected loci: Out of 31,923 peaks in DPY30 knockdown hESCs, 6482 (20.3%) were decreased, and 1394 (4.4%) were increased, while 4028 (12.3%) and 1510 (4.6%) of the 32,642 peaks in NANOG knockdown hESCs were decreased and increased, respectively (Supplemental Fig. S6B; Supplemental Table S5). Interestingly, regions with decreased H3K4me3 after DPY30 or NANOG knockdown were associated with genes involved in the development of tissues from all germ layers (Supplemental Table S5), suggesting a role for DPY30 and NANOG in the regulation of developmental processes. Importantly, we observed a large overlap in the H3K4me3 peaks impaired after DPY30 or NANOG knockdown, implying that these two factors control a large set of common genes (2062 peaks using our conservative analysis thresholds: *P*-value, 1.00 × 10^−6^; FDR, 0.1%; fold change, >1.5 [Fig. 6A]; and 4071 peaks with less stringent thresholds: *P*-value, 1.00 × 10^−4^; FDR, 1%; fold change, >1.25 [Supplemental Fig. S6C]). Nevertheless, we observed a number of regions specifically affected by either protein, which was particularly large for DPY30 (4336 and 6209 peaks, respectively, for the two analyses), in agreement with its general role in promoting the activity of COMPASS complexes (Jiang et al. 2011). These observations demonstrated that NANOG and DPY30 are required to maintain H3K4me3 on a large but specific subset of genomic regions involved in early cell fate decisions.

Interestingly, a large proportion (44%) of H3K4me3 peaks decreasing after 2 h of SB significantly overlapped with those decreasing after both NANOG and DPY30 knockdown (Fig. 6B, overlap *Z*-score of 30.63, *P* = 4.11 × 10^−206^, as calculated with MULTOVL; see also Supplemental Fig. S6D for the same analysis with less stringent thresholds: *P*-value, 1.00 × 10^−4^; FDR 1%; fold change, >1.25). These regions were associated to genes that include the key pluripotency markers *NANOG*, *POU5F1/OCT4*, *DPPA4*, and *PRDM14* and the mesendoderm regulators *LEFTY1*, *WNT3*, *CER1*, *FGF8* , and *NODAL* (Fig. 6C). Therefore, while the long-term knockdown of NANOG and DPY30 induces a larger H3K4me3 deregulation compared with the one resulting from 2 h of inhibition of Activin/Nodal signaling, there appears to be a core set of crucial genes that are directly controlled by SMAD2/3, NANOG, and DPY30.

On the other hand, the large number of H3K4me3 peaks that decreased by >50% after both DPY30 knockdown and NANOG knockdown but not after 2 h of SB (1847 peaks) could be controlled independently of SMAD2/3. However, the expression of several of these genes was significantly impaired after 48 h of inhibition of Activin/Nodal signaling with SB (Fig. 6D), arguing against a SMAD2/3-independent regulation. Furthermore, there was still a significant decrease in H3K4me3 after SB for 2 h on these genomic regions (Fig. 6E), but this effect was not uncovered by our previous analysis, which filtered results for only major modifications (>50%). The observation that H3K4me3 of these regions was modestly affected after 2 h of SB suggested that they might represent a class of genes that have a slower kinetics of H3K4me3 loss. This was supported by ChIP-qPCR for several such genes showing that the decrease of H3K4me3 reached its maximum only after 48 h of Activin/Nodal inhibition (Fig. 6F). In conclusion, there is a large number of genes where H3K4me3 is controlled by SMAD2/3, DPY30, and NANOG, but the full histone demethylation on these targets occurs in a slow fashion and only after chronic inhibition of Activin/Nodal.

In addition to H3K4me3, we also performed ChIP-seq for H3K27me3 after DPY30 knockdown or NANOG knockdown (Supplemental Fig. S6A,B; Supplemental Table S5). In sharp contrast to the results from treatment with SB for 2 h, we identified many regions of differential H3K27me3 (2669 decreased and 2240 increased regions out of 12,728 peaks for DPY30 knockdown; 4580 decreased and 3333 increased regions out of 16,152 peaks for NANOG knockdown). Moreover, the regions in which H3K27me3 changed after DPY30 or NANOG knockdown did not show any significant difference in the H3K27me3 levels after 2 h of SB (Supplemental Fig. S6E). These data implied that this repressive histone mark required more time to change when compared with H3K4me3. This suggests that Activin/Nodal signaling might not be directly involved in the regulation of H3K27me3 in hESCs, while NANOG and DPY30 could have a more important role in this process. In summary, these results confirmed and validated a model in which SMAD2/3, NANOG, and DPY30 collaborate to maintain H3K4me3 on a subset of genes, which can be divided into two classes defined by the speed at which they lose H3K4me3, thereby suggesting an epigenetic hierarchy between different regulators of cell fate decisions.

### DPY30, NANOG, and SMAD2/3 colocalize at the genome*-*wide level

We next decided to investigate whether the functional interactions described above could imply collaborative mechanisms between SMAD2/3, NANOG, and DPY30 at a genome-wide level. For that, we performed ChIP-seq for DPY30 on control (SCR shRNA) hESCs and DPY30 knockdown hESCs. These analyses revealed that DPY30 could be found on 26,387 genomic regions in hESCs, while only 6893 peaks were identified in DPY30 knockdown hESCs (Supplemental Table S5). Moreover, DPY30 ChIP-seq coverage was strongly impaired in DPY30 knockdown hESCs compared with control hESCs (Supplemental Fig. S6F,G). Finally, DPY30 binding correlated with H3K4me3 deposition, as expected and previously shown for mESCs (Supplemental Fig. S6F; Jiang et al. 2011). Overall, these findings confirmed the specificity of the DPY30 antibody and validated the quality of the DPY30 ChIP-seq. We then combined this data set with pre-existing ChIP-seq analyses for SMAD2/3 (Brown et al. 2011) and NANOG (ENCODE data) (Bernstein et al. 2012). Interestingly, a large proportion of genome-wide binding sites of SMAD2/3 were co-occupied by both NANOG and DPY30 read enrichment (Supplemental Fig. S6H,I). Of note, the colocalization of SMAD2/3 and NANOG is in agreement with previous reports (Mullen et al. 2011), while our data suggest that DPY30 is also present in this complex. Interestingly, NANOG- and DPY30-binding sites overlapped or were closely associated to SMAD2/3-binding sites located on key genes on which H3K4me3 is decreased after 2 h of SB (Fig. 6G). Furthermore, NANOG and DPY30 extensively co-occupied SMAD2/3-binding sites associated with H3K4me3 regions responsive to Activin/Nodal signaling (located 100 kb upstream/downstream, as described earlier) (Fig. 6H), and the extent of NANOG and DPY30 enrichment on such regions was even higher than what was observed genome-wide on all SMAD2/3-binding sites (Fig. 6I; Supplemental Fig. S6I). Overall, these findings demonstrated that SMAD2/3, NANOG, and DPY30 can be found in close proximity at the genome-wide level, thereby reinforcing our hypothesis that these factors form a complex controlling H3K4me3 deposition on target genes regulated by Activin/Nodal signaling.

### Dpy30 is necessary to maintain the pluripotent state of the post-implantation epiblast

**Figure 7.**
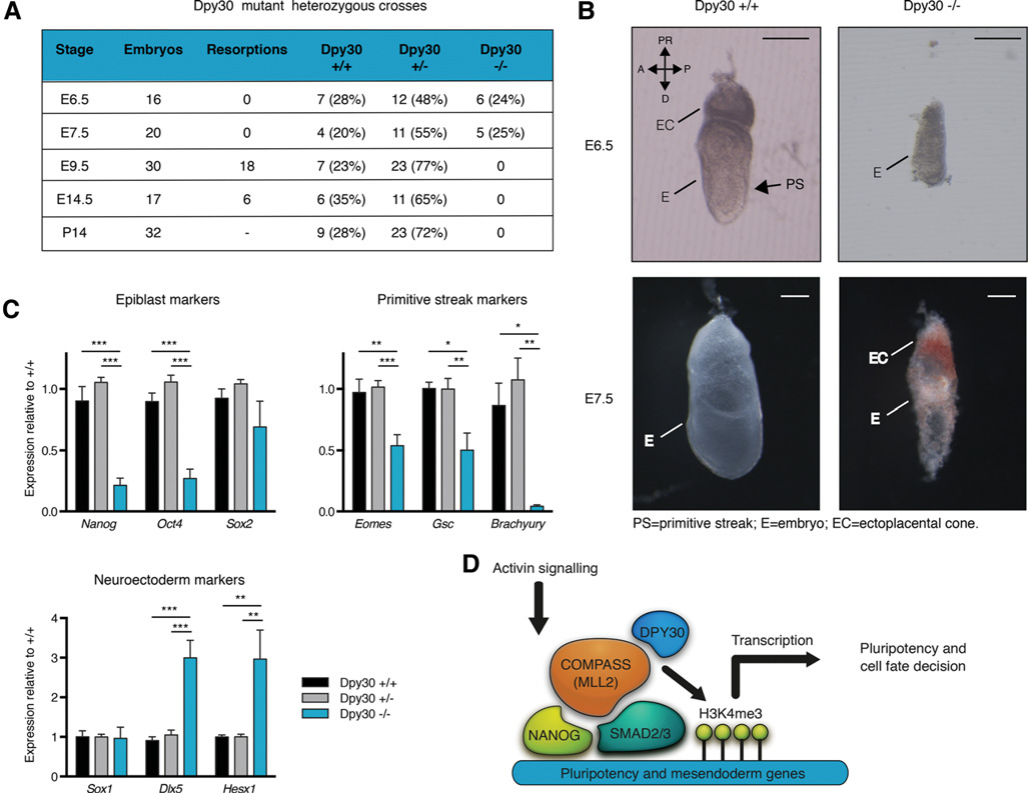
Dpy30 is required for mouse post-implantation embryonic development. (*A*) Genotyping results from Dpy30 mutant heterozygous crosses. Stages are embryonic (E) or postnatal (P) days. (*B*) Bright-field images of wild-type (+/+) or Dpy30 knockout (−/−) embryos at E6.5 or E7.5. The anterior–posterior (A/P) and proximal–distal (PR/D) axes are shown. Bars, 100 μm. (*C*) Gene expression qPCR in E6.5 embryos from Dpy30 mutant heterozygous crosses. Note that *Sox2* is both an epiblast and a neuroectoderm marker. Significant differences versus Dpy30^+/+^ as calculated by one-way ANOVA are reported. *n* = 6 for Dpy30^+/+^ and Dpy30^−/−^; *n* = 11 for Dpy30^+/−^. (*D*) Schematic of the model that we propose for Activin/Nodal-dependent transcriptional regulation of pluripotency and mesendoderm genes in hESCs and mouse epiblast cells. Note that the interactions depicted in the model must be interpreted as functional ones rather than direct protein–protein interactions, as this aspect was not the focus of the present study.

To confirm the relevance of the mechanisms uncovered by our studies in vitro, we decided to evaluate the function of Dpy30 during embryonic development in mice. For that, we took advantage of Dpy30 knockout mice generated by the Mouse Genetic Program of the Wellcome Trust Sanger Institute [Dpy30<tm1a(KOMP)Wtsi>] (Supplemental Fig. S7A–C). Mice carrying heterozygous mutations were healthy, and we did not observe any obvious phenotype. However, viable homozygous mutant mice could never be recovered, and further analysis revealed that the absence of Dpy30 was embryonic-lethal between embryonic day 7.5 (E7.5) and E9.5 (Fig. 7A). In addition, mutant embryos recovered at E6.5 displayed gross morphological abnormalities, including reduced size and no clear anterior–posterior patterning, as marked by the absence of the primitive streak (Fig. 7B). Accordingly, Dpy30 knockout embryos at E7.5 were developmentally delayed and underwent resorption (Fig. 7B). Importantly, the absence of *Dpy30* expression in homozygous mutant embryos was confirmed by qPCR to validate the gene targeting strategy (Supplemental Fig. S7D). Moreover, Dpy30 knockout embryos dissected at E6.5 showed prematurely reduced expression of epiblast markers (*Nanog* and *Pou5f1/Oct4*) and failed to properly induce mesendoderm genes (*Eomes*, *Gsc*, and *Brachyury*), while neuroectoderm markers were either unaffected (*Sox2* and *Sox1*) or even increased (*Dlx5* and *Hesx1*) (Fig. 7C). Therefore, we concluded that Dpy30 is necessary to maintain the pluripotent state of the epiblast of the post-implantation embryo and also enable proper specification of the three germ layers in vivo. These results strikingly recapitulate the phenotype induced by the knockdown of DPY30 in hESCs, indicating that the molecular regulations that we uncovered in vitro could also occur at the corresponding developmental stage during early embryogenesis (Fig. 7D).

## Discussion

Here we uncovered the mechanisms by which extracellular signals are converted into epigenetic and transcriptional regulations necessary for hESC pluripotency and cell fate decisions. The effectors of the Activin/Nodal signaling pathway SMAD2/3 and NANOG recruit DPY30-containing COMPASS complexes onto specific genomic regions where they maintain H3K4me3. This event is essential to maintain the expression of a core set of factors necessary for pluripotency. Accordingly, inhibition of Activin/Nodal signaling results in a rapid decrease in both H3K4me3 and expression of several master regulators of hESC pluripotency, including NANOG itself. NANOG is involved in a feed-forward regulatory network that maintains its own expression and that of other core pluripotency factors, such as OCT4, all of which are part of a broader SMAD2/3-dependent transcriptional network characterizing hPSCs (Mullen et al. 2011; Teo et al. 2011). As such, the quick loss of H3K4me3 upon Activin/Nodal inhibition provides hESCs with an extremely rapid way to switch off the expression of master pluripotency regulators, including NANOG. This mechanism initiates a cascade of transcriptional network modifications necessary for timely and efficient cell fate decisions.

Our data show that MLL2/KMT2B could be the COMPASS complex regulating a broad part of the transcriptional network downstream from Activin/Nodal signaling. Interestingly, MLL2/KMT2B was reported to regulate H3K4me3 on bivalent promoters in mESCs (Hu et al. 2013; Denissov et al. 2014). Nonetheless, we cannot exclude that other COMPASS complexes are involved in these regulations in hESCs, and further investigation will be necessary to explore this possibility. Similarly, additional epigenetic modifiers could be involved in the regulations uncovered by our study, since DPY30 has been shown to also interact with the NURF complex (van Nuland et al. 2013). However, we could not detect any signaling-dependent binding event of the NURF complex on Activin/Nodal genes, suggesting that the activity of this complex is not directly relevant for the mechanisms discussed here.

Interestingly, the prompt and selective decrease of H3K4me3 on pluripotency genes upon signaling inhibition suggests the existence of an active process to erase positive histone marks on these specific genomic regions. Likely candidates for such a role are the H3K4-specific histone demethylases of the JARID1 family. Indeed, the profile of histone methylation after inhibition of Activin/Nodal signaling is consistent with the sequential H3K4me3 and H3K4me2 demethylation that is known to be mediated by this class of demethylases (Cloos et al. 2008). Interestingly, knockdown in Jarid1B expression in mESCs impairs silencing of pluripotency genes and differentiation into neuroectoderm (Schmitz et al. 2011). Therefore, the epigenetic status of a core pluripotency network could be tightly controlled by extracellular signals through the dynamic competition of histone methylation writers and erasers.

Our results also show that Activin/Nodal signaling controls H3K4me3 presence on “bivalent genes” known to be master regulators of mesendoderm specification. Inhibition of Activin/Nodal, NANOG knockdown, and DPY30 knockdown are all associated with a decrease of H3K4me3 on these loci, which correlates with an impaired capacity of hESCs to differentiate toward mesendoderm. These results suggest that positive marks are maintained by active mechanisms on key bivalent genes and that the deposition of these marks could be indispensable for proper induction of differentiation. This supports the hypothesis that bivalent marks are necessary to poise developmental genes for efficient and synchronous cell fate decisions in response to extracellular signaling (Bernstein et al. 2006; Vastenhouw and Schier 2012).

Importantly, our study suggests that the presence of H3K4me3 on Activin/Nodal target genes not only is a consequence of the level of transcription but might also be a causal event that directly influences gene expression. Indeed, knockdown of the epigenetic remodeler DPY30 is sufficient to mimic the transcriptional effect of Activin/Nodal inhibition. Several epigenetic readers are known to link H3K4me3 with various factors that can promote gene expression, including TFIID, SAGA, NuRF/BPTF, and Sin3B/HDAC complexes (Vermeulen et al. 2010). Thus, it is tempting to assume that H3K4me3 deposition could control transcription trough these interactions. However, in-depth functional analyses will be necessary to confirm this hypothesis linking loss of H3K4me3 and decreased transcription of Activin/Nodal target genes in hESCs.

The role of H3K4me3 in pluripotency and differentiation has been a matter of open controversy (Vastenhouw and Schier 2012; Voigt et al. 2013). Studies in mESCs showed that knockdown of Dpy30 or Rbbp5 impairs only differentiation without affecting the expression of pluripotency genes, while knockdown of Wdr5 results in dramatic loss of self-renewal (Ang et al. 2011; Jiang et al. 2011). These diverging results might arise from either different levels of H3K4me3 impairment or the known pleiotropic functions of Wdr5 (van Nuland et al. 2013). Importantly, these studies were limited to in vitro experiments in mESCs and hence did not test the relevance of H3K4me3 during in vivo development or provide evidence for an evolutionarily conserved role of H3K4me3 across mice and humans. Our study addresses both of these aspects: By combining both experiments in hESCs and in vivo analyses in mice, we demonstrated an evolutionarily conserved fundamental role of H3K4me3 in pluripotency and cell fate decisions. The divergent function of DPY30 in hESCs and mESCs can be explained by their different embryonic identities. Indeed, hESCs are phenotypically related to the PSCs of the post-implantation epiblast, while mESCs resemble cells of the inner cell mass (ICM) (Brons et al. 2007; Vallier et al. 2009c). This hypothesis is supported by our genetic study in mice showing that Dpy30 is essential only for post-implantation development and gastrulation of the epiblast. Thus, H3K4me3 deposition could be of prominent importance specifically during the establishment of the epiblast, when Activin/Nodal signaling starts to orchestrate the core pluripotency network. It is also worth highlighting that the Dpy30 knockout mouse phenotype closely recapitulates the one of embryos mutant for Nodal or Smad2/3 (Brennan et al. 2001; Camus et al. 2006). Indeed, the absence of Nodal signaling results in the loss of pluripotency markers in the epiblast, impaired gastrulation, and ectopic expression of neuroectoderm markers. Therefore, epigenetic control imposed by Activin/Nodal signaling in hESCs is also taking place in the late epiblast during embryonic development. Overall, our results show that the function of epigenetic modifiers can significantly differ in the pre- and post-implantation pluripotent embryos and thus should be carefully evaluated using different types of PSCs that resemble these different developmental stages.

The interconnection between Activin/Nodal signaling and SMAD2/3-dependent deposition of H3K4me3 on master regulators of cell fate could be relevant for many cell types. Indeed, TGFβ is a widespread pathway (Massagué 2012), and H3K4me3 is a histone mark that is deposited in virtually any cell type (Ruthenburg et al. 2007). Of particular interest, TGFβ signaling regulates the balance between self-renewal and differentiation of several adult stem cells as well as many cancer stem cells (Caja et al. 2012). Thus, our findings not only reveal the mechanisms that coordinate Activin/Nodal signaling with epigenetic and transcriptional networks during early cell fate decisions of hESCs but also establish general principles that can be applicable to stem cells involved in human development and disease.

## Materials and methods

### hESC culture, differentiation, and characterization

Feeder-free H9 hESCs (WiCell) were grown in chemically defined culture medium with 10 ng/mL Activin A and 12 ng/mL FGF2 as described in Brons et al. (2007). The formulation of all cell culture media and reagents is reported in Supplemental Table S6. Activin/Nodal inhibition was performed by replacing Activin A with 10 μM SB. hESCs were differentiated into endoderm for 3 d in CDM-PVA with 20 ng/mL FGF2, 10 μM Ly-294002, 100 ng/mL Activin A, and 10 ng/mL BMP4, while neuroectoderm differentiation was induced for 6 d in CDM with 12 ng/mL FGF2 and 10 μM SB (Vallier et al. 2009b). Pancreatic and hepatic specification was initiated after endoderm differentiation, and details of these differentiation protocols can be found in Supplemental Table S6. Teratomas were induced by injection of 1 million hESCs into the testicular lumen of SCID mice and analyzed after 12 wk by hematoxylin and eosin staining. Transient and stable knockdowns were performed using pLKO.1 plasmids with specific shRNAs transfected with Lipofectamine 2000 as described in Teo et al. (2011).

### Gene and protein expression analysis

Standard methods for qPCR, immunostaining, Western blot, and flow cytometry were previously described (Vallier et al. 2009b; Pauklin and Vallier 2013), and details on primers and antibodies used are in Supplemental Table S6. GraphPad Prism 5 was used for statistical analysis. One-way or two-way ANOVA tests were followed by Bonferroni's corrected multiple comparisons between pairs of conditions. Unless otherwise indicated in the figure legends, we analyzed three biological replicates for each data point in all graphs, and the level of significance was as follows: *P* < 0.05 (*), *P* < 0.01 (**), and *P* < 0.001 (***).

### Immunoprecipitation and ChIP

Immunoprecipitation and ChIP and were performed as described previously in Brown et al. (2011) and Pauklin and Vallier (2013), and details on protocols, reagents, antibodies, and genomic primers are reported in the Supplemental Material and Supplemental Table S6. ChIP-qPCR data were normalized on a negative control region (transcription factor ChIP) or the amount of input DNA (histone mark ChIP). Sequential ChIP was performed as described in Truax and Greer (2012) with minor modifications as described in the Supplemental Material.

### Microarrays

Microarrays were performed on biological triplicates using Illumina HT12-v4.0 bead chips, and the raw data were processed using Genome Studio (Illumina) and normalized using the R/Bioconductor package lumi. The raw and processed microarray data are available on ArrayExpress (E-MTAB-2749). Microarray bioinformatic analyses are described in the Supplemental Material.

### ChIP-seq

ChIP samples were sequenced using Illumina HiSeq 2000, and the raw data are publicly available on ArrayExpress (E-ERAD-191: histone mark ChIP-seq; E-ERAD-365: DPY30 ChIP-seq). ChIP-seq bioinformatic analyses are described in the Supplemental Material.

### Generation and phenotyping of *Dpy30* knockout mice

All animal procedures were performed in agreement with the United Kingdom Home Office regulations, United Kingdom Animals (Scientific Procedures) Act of 1996. *Dpy30* knockout mice were generated by the Sanger Institute Mouse Genetics Project. Dysmorphology assessments were performed on embryos dissected from the decidua of pregnant females after removal of extraembryonic tissues, and qPCR analyses were done on RNA extracted from individual embryos.

Detailed descriptions and additional references for all the experimental and bioinformatics procedures presented here are provided in the Supplemental Material.

## Supplementary Material

Supplemental Material
