# Supplementary material for: Activin/Nodal signaling and NANOG orchestrate human embryonic stem cell fate decisions by controlling the H3K4me3 chromatin mark
Source: Genes Dev. 2015 Apr 1;29(7):702–17. doi: 10.1101/gad.255984.114 (PMC4387713; doi:10.1101/gad.255984.114)
Supplement: Supplemental Material [file supp_29_7_702__index.html]

Supplemental Material 

# Activin/Nodal signaling and NANOG orchestrate human embryonic stem cell fate decisions by controlling the H3K4me3 chromatin mark

## Supplemental Material

**Files in this Data Supplement:**

- Supplemental Information.pdf
- Table S1.xlsx
- Table S2.xlsx
- Table S3.xlsx
- Table S4.xlsx
- Table S5.xlsx
- Table S6.xlsx
