# Supplementary material for: Activin/Nodal signaling and NANOG orchestrate human embryonic stem cell fate decisions by controlling the H3K4me3 chromatin mark
Source: Genes Dev. 2015 Apr 1;29(7):702–17. doi: 10.1101/gad.255984.114 (PMC4387713; doi:10.1101/gad.255984.114)
Supplement: Supplemental Material [file supp_gad.255984.114_Supplemental_Information.pdf]

SUPPLEMENTAL MATERIAL for

Activin/Nodal Signalling and NANOG Orchestrate Human Embryonic Stem Cell Fate Decisions by Controlling the H3K4me3 Chromatin Mark

Bertero A. et al.

LIST OF CONTENTS:

Supplemental Figures (pag. 3-16)

- Supplemental Figure 1, related to Figure 1 and Table S1. Activin/Nodal regulates H3K4me3 of a subset of genes in hESCs.
- Supplemental Figure 2, related to Figure 2 and Table S2. Dynamics of the transcriptional response to Activin/Nodal inhibition and their relationship with epigenetic changes
- Supplemental Figure 3, related to Figure 3 and Table S3. DPY30 is required for H3K4me3 and expression of SMAD2/3 target genes.
- Supplemental Figure 4, related to Figure 4. DPY30 is required for mesendoderm differentiation of hESCs.
- Supplemental Figure 5, related to Figure 5 and Table S4. SMAD2/3 and NANOG recruit DPY30 onto their genomic targets.
- Supplemental Figure 6, related to Figure 6 and Table S5. SMAD2/3, NANOG and DPY30 control H3K4me3 on a subset of shared genes.
- Supplemental Figure 7, related to Figure 7. Dpy30 is required for mouse post-implantation embryonic development.

Supplemental Tables (online)

- Supplemental Table 1, related to Figure 1 and Figure S1. List of H3K4me3 and H3K27me3 peaks up or down-regulated after inhibition of Activin/Nodal signalling.
- Supplemental Table 2, related to Figure 2 and Figure S2. List of genes differentially expressed after different time points of Activin/Nodal signalling inhibition.
- Supplemental Table 3, related to Figure 3 and Figure S3. List of genes differentially expressed after knock-down of DPY30 in hESCs.
- Supplemental Table 4, related to Figure 5 and Figure S5. List of genes differentially expressed after knock-down of NANOG in hESCs.
- Supplemental Table 5, related to Figure 6 and Figure S6. List of H3K4me3 and H3K27me3 peaks up or down-regulated after knock-down of DPY30 or NANOG in hESCs, and list of DPY30 peaks in control or DPY30 knock-down hESCs.
- Supplemental Table 6, related to Material and Methods and Supplemental Material and Methods. Lists of all reagents used for the study.

Supplemental Material and Methods (pag. 17-32)

Supplemental References (pag. 33-35)

SUPPLEMENTAL FIGURES

Bertero_FigS1

A

All decreased H3K4me3 peaks

GREAT analysis - Pathway commons

Term	Rank	-log10 pval
Regulation of signalling by Nodal	2	3.84
Wnt signalling network	4	3.7

GREAT analysis - MGI expression

Term	Rank	-log10 pval
TS6 - Epiblast	1	6.72
TS9 - Endoderm	4	4.55

B

Decreased H3K4me3 peaks on bivalent promoters

GREAT analysis - MGI Expression

Term	Rank	-log10 pval
TS9 - Primitive Streak	2	6.43
TS10 - Mesoderm	6	5.93
TS9 - Endoderm	7	5.75

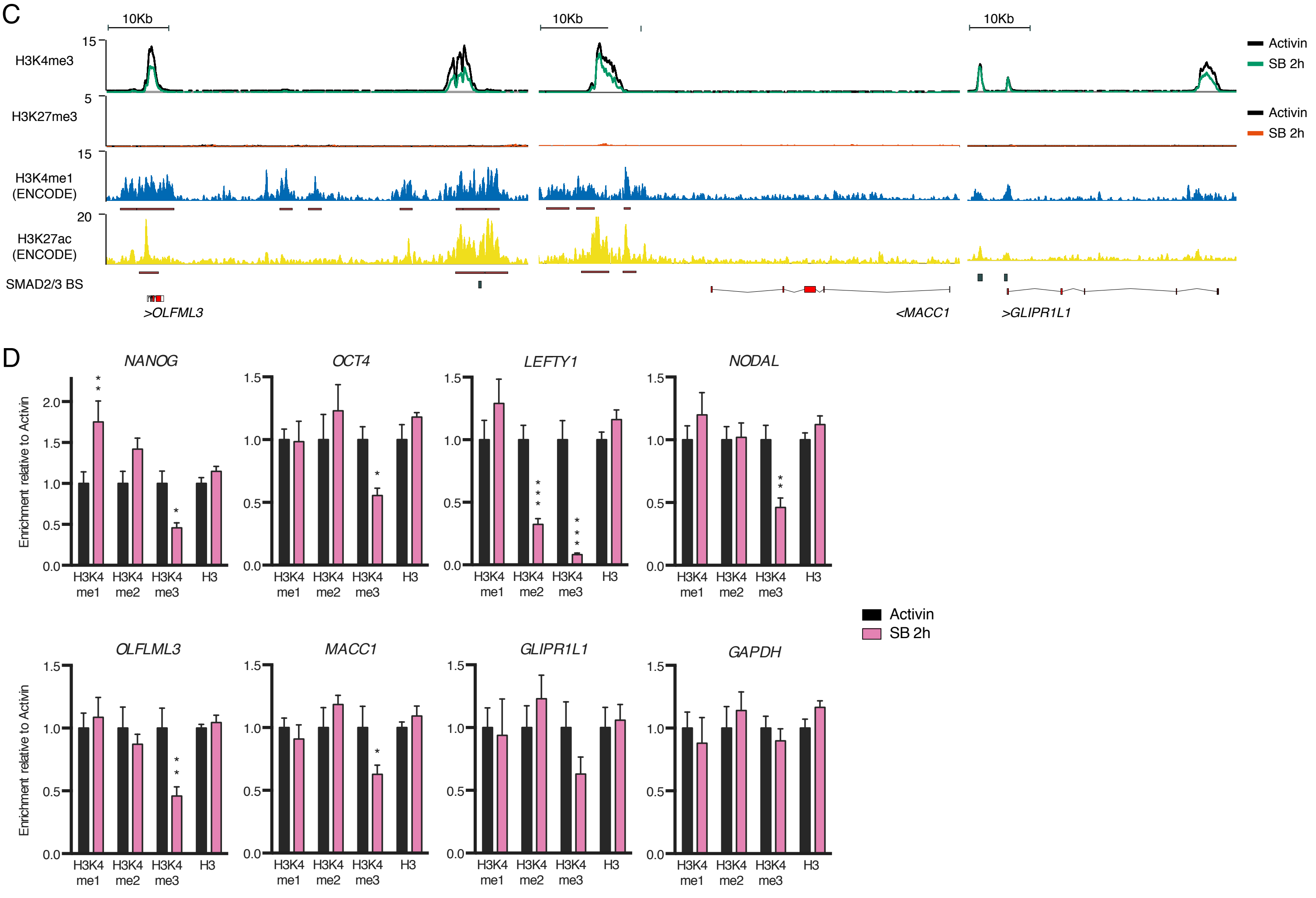

Figure S1, related to Figure 1 and Table S1. Activin/Nodal regulates H3K4me3 of a subset of genes in hESCs. (A) Selected results of GREAT analysis (McLean et al. 2010) on the H3K4me3 peaks downregulated after inhibition of Activin/Nodal signalling for 2h with SB. (B) As in A, but the analysis was performed only on H3K4me3 peaks in a range of ± 5Kb from the closest TSS and co-localizing with H3K27me3 marks (bivalent

promoters). The full results both GREAT analyses are in Table S1. (C) ChIP-seq results for H3K4me3 and H3K27me3 on selected representative intergenic H3K4me3 peaks before and after inhibition of Activin with SB for 2h. Lines represent read-enrichments normalized by million mapped reads and size of the library. H3K4me1 and H3K27ac ChIP-seq data and peak calls (below) for the H1 hESCs line from the ENCODE project are shown. SMAD2/3 binding sites in hESCs (from Brown et al., 2011) are reported. (D) ChIP-qPCR for H3, H3K4me1, H3K4me2 and H3K4me3 before and after 2h of SB. qPCR were performed against the genomic regions showing decreased H3K4me3 in Fig. 1E and S1C. Significant differences vs Activin for each gene as calculated by t-tests are reported.

Bertero_FigS2

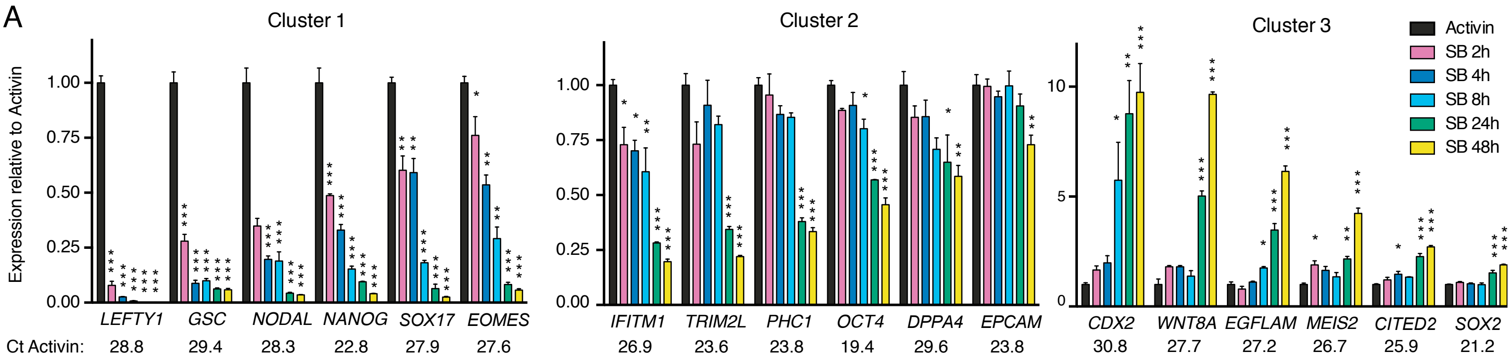

B

Expression change of genes closest to H3K4me3 peaks decreased after 2h SB		
Friedman's statistic 151.4 (p <0.0001)		
Comparison	Rank sum diff.	Adj. P-val
Activin vs SB 2h	173.5	0.0847
Activin vs SB 4h	282.5	0.0001
Activin vs SB 8h	428.5	<0.0001
Activin vs SB 24h	566.0	<0.0001
Activin vs SB 48h	643.5	<0.0001

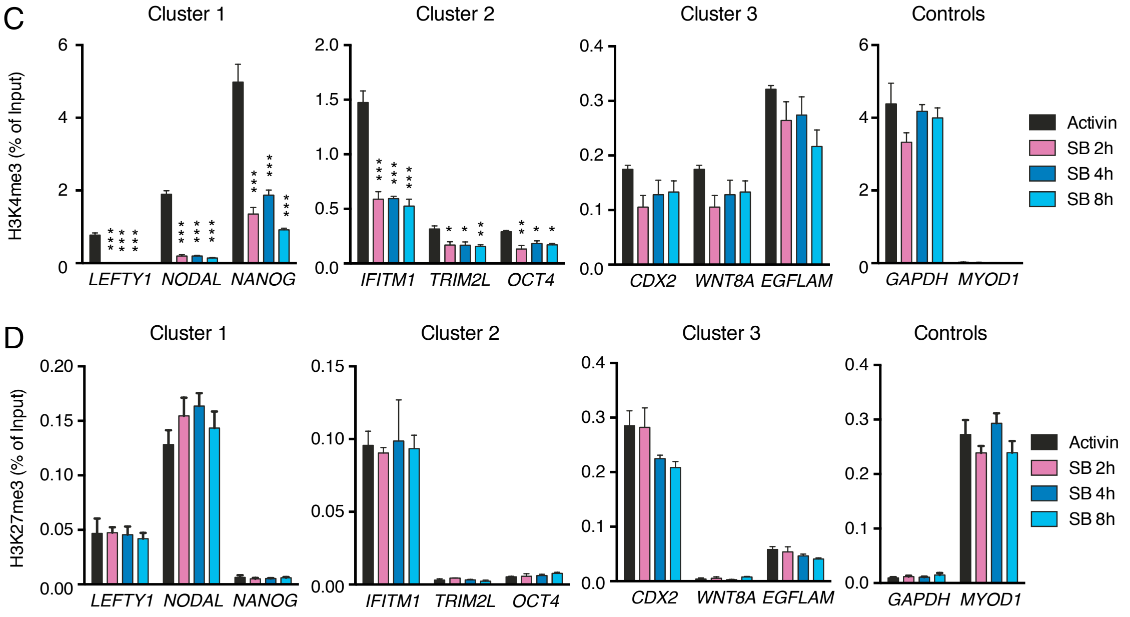

Figure S2, related to Figure 2 and Table S2. Dynamics of the transcriptional response to Activin/Nodal inhibition and their relationship with epigenetic changes. (A) Gene expression qPCR before and after inhibition of Activin with SB for 2h, 4h, 8h, 24h, and 48h. Gene clusters refer to those described in Fig. 2B and 2C. The average cycle threshold (Ct) for each gene in hESCs cultured in the presence of Activin is reported (average Ct for the housekeeping gene *HMBS/PBGD* was 25.02). Significant differences vs Activin for each gene as calculated by one-way ANOVA are reported. (B) Statistical analysis of the changes in expression after SB treatment of the genes closest to the H3K4me3 peaks decreased after 2h of SB. The overall significance of changes across the time-course (Friedman's statistic) and the significance of paired comparisons for each time point vs Activin are reported (Dunn's multiple comparisons test). (C) ChIP-qPCR for H3K4me3 on SMAD2/3 target genes before and after SB for 2h, 4h or 8h. Gene clusters refer to those from Fig. 2B and 2C. *GAPDH*

and *MYOD1* are positive and negative controls for the ChIP. Significant differences vs Activin for each gene as calculated by one-way ANOVA are reported. (D) ChIP-qPCR as in B, but for H3K27me3. *GAPDH* and *MYOD1* are negative and positive controls for the ChIP.

Bertero_FigS3

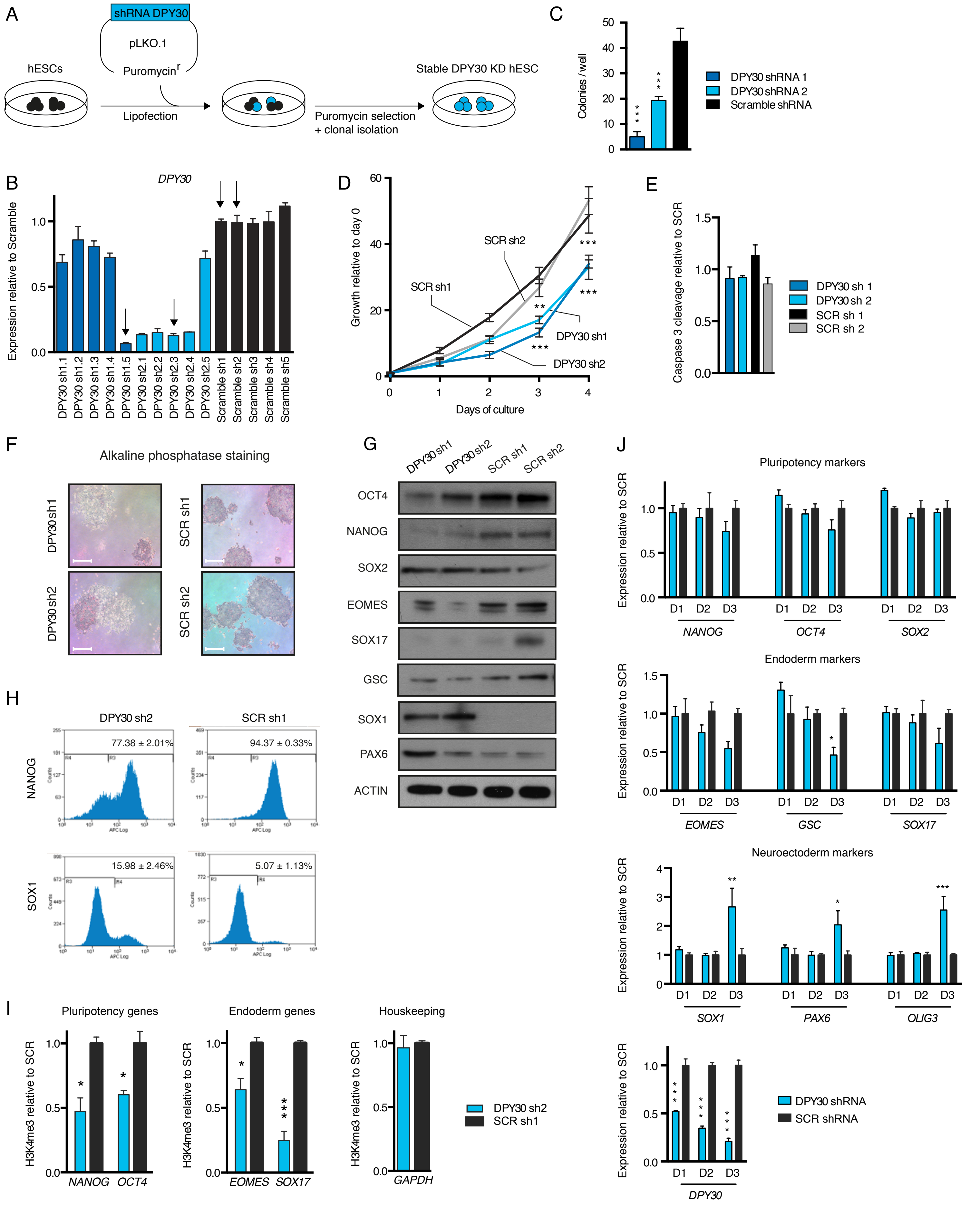

Figure S3, related to Figure 3 and Table S3. DPY30 is required for H3K4me3 and expression of SMAD2/3 target genes. (A) Schematics of the generation of stable DPY30 KD hESCs. (B) qPCR for *DPY30* in hESCs clonal lines expressing two different DPY30 shRNAs (sh1 or sh2) or a scramble shRNA (five clones per condition). Arrows indicate the clones selected for further experiments. (C) Number of total colonies obtained after selection of stable integrants for two different DPY30 shRNAs or a scramble shRNA. Significant differences vs SCR shRNA as calculated by one-way ANOVA are reported. (D) Growth curve for DPY30 KD or control hESCs. Significant differences vs both SCR sh1 and SCR sh2 (only the highest p is shown) as calculated by two-ways ANOVA are reported; n=4 biological replicates. (E) Apoptotic index (measured by Caspase-3 cleavage assay) of DPY30 KD or control hESCs. No significant differences vs both SCR sh1 and SCR sh2 as calculated by one-way ANOVA were identified. (F) Alkaline phosphatase staining of DPY30 KD or control hESCs. Positive staining is in purple/blue. Scale bars are 200μm (G) Western blots in DPY30 KD and control hESCs. (H) Representative flow-cytometry histograms in DPY30 KD or control hESCs. The gates used to define positive cells are shown, and the average percentages ± SEM of positive cells from three biological replicates are reported. (I) ChIP-qPCR for H3K4me3 in DPY30 KD and control hESCs. For each gene, significant differences vs SCR as calculated by t-test are reported. (J) Gene expression qPCR in hESCs transiently transfected with an shRNA against DPY30 or a control shRNA. Samples were collected after 24h, 48h, and 72h from the transfection (D1, D2 and D3 respectively). Note that *SOX2* is both a pluripotency and a neuroectoderm marker. For each gene, significant differences vs SCR as calculated by two-ways ANOVA are reported.

Bertero_FigS4

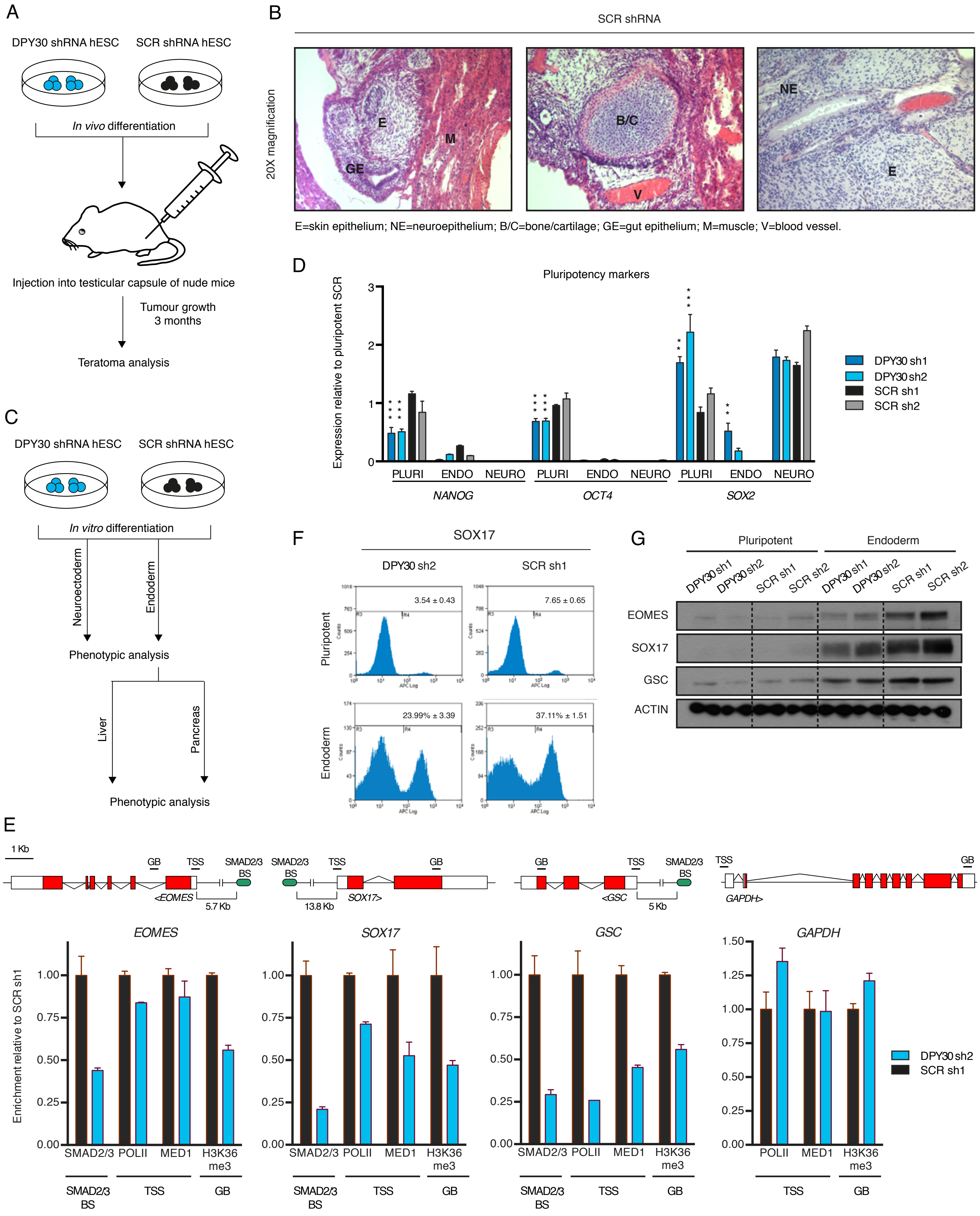

Figure S4, related to Figure 4. DPY30 is required for mesendoderm differentiation of hESCs. (A) Schematics of the induction of teratomas from DPY30 KD or control hESCs. (B) Hematoxilin and eosin histological stainings of teratomas derived from SCR control hESCs. (C) Schematics of the *in vitro* directed differentiation of DPY30 KD or control hESCs. (D) Gene expression qPCR in DPY30 KD or control hESCs before (PLURI) or after *in vitro* directed differentiation towards endoderm or neuroectoderm. Note that *SOX2* is both a pluripotency and a neuroectoderm marker. For each gene, significant differences vs both SCR sh1 and SCR sh2 in the same condition (only the highest p is shown) as calculated by two-way ANOVA are reported. (E) ChIP-qPCR for SMAD2/3, RNA polymerase II (POLII) or Mediator (MED1) in DPY30 KD or control hESCs after endoderm differentiation. The location of the primers used for qPCR is indicated in the schematic above the graphs (TSS: transcription start site; GB: gene body; SMAD2/3 BS: SMAD2/3 binding site). The results are representative of three independent experiments (F) Representative flow-cytometry histograms for SOX17 in DPY30 KD or control hESCs before (pluripotent) or after endoderm differentiation. The gates used to define positive cells are shown, and the average percentages ± SEM of positive cells from three biological replicates are reported. (G) Western blots in DPY30 KD or control hESCs before (pluripotent) or after endoderm differentiation.

Bertero_FigS5

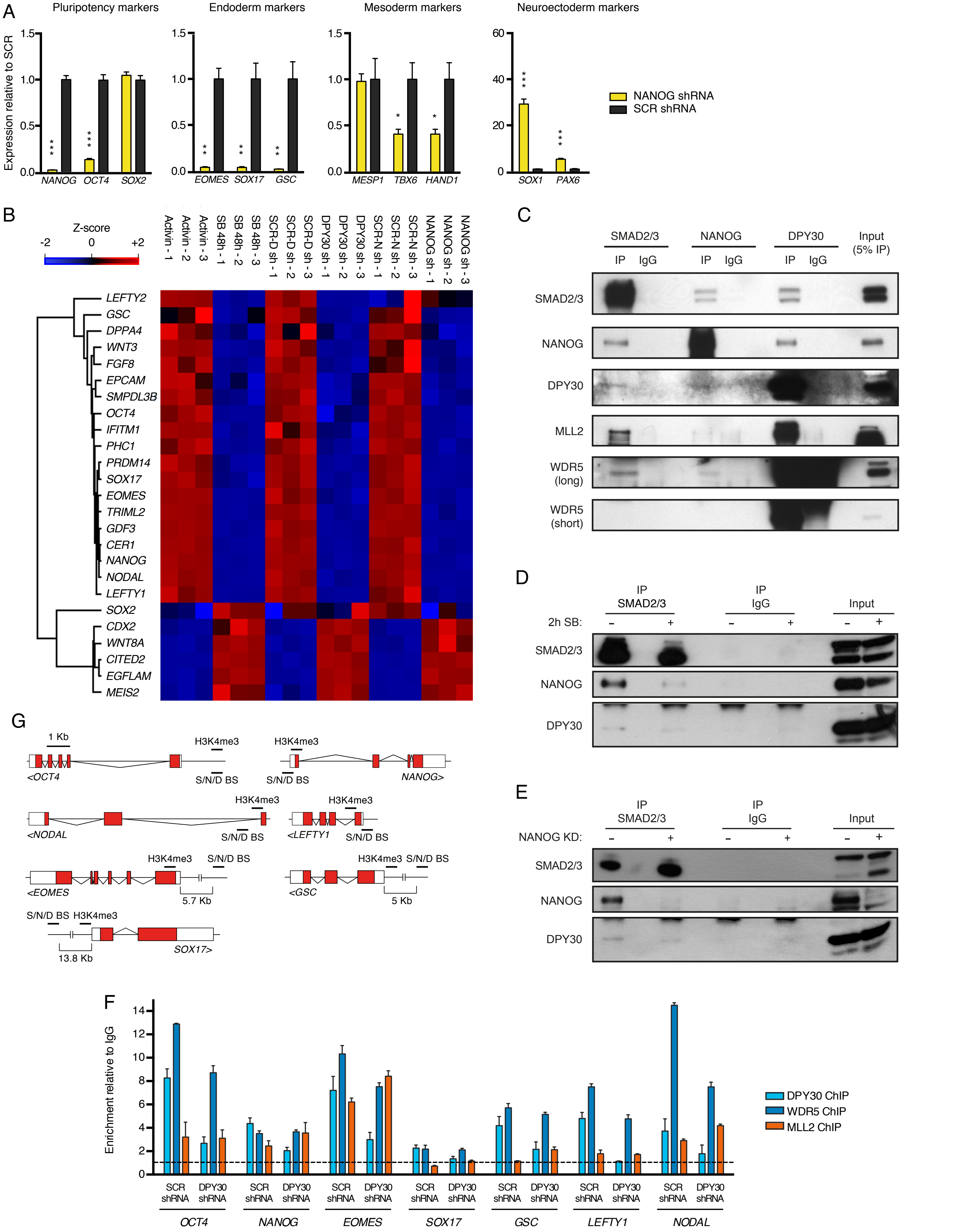

Figure S5, related to Figure 5 and Table S4. SMAD2/3 and NANOG recruit DPY30 onto their genomic targets. (A) Gene expression qPCR in NANOG KD and control hESCs. Note that *SOX2* is both a pluripotency and a neuroectoderm marker. For each gene, significant differences vs SCR as calculated by t-tests are reported. (B) Heatmap reporting changes in gene expression of selected SMAD2/3 target genes after NANOG KD, DPY30 KD and 48h of SB. Z-scores were separately calculated for each experiment. (C) Western blots of SMAD2/3, NANOG, DPY30 or control (IgG) immunoprecipitations from nuclear extracts of hESCs. Input is 5% of the material used for IP. Note that the high level of background for the WDR5 blot in the DPY30 IP and matched rabbit IgG control is due to the reactivity of the anti-rabbit secondary antibody against the rabbit immunoglobulins used for the IP. A short exposure that shows WDR5 binding to DPY30 is provided. (D) Western blots of SMAD2/3 or control (IgG) immunoprecipitations from nuclear extracts of hESCs before or after inhibition of Activin/Nodal for 2h with SB. Input is 5% of the material used for IP. (E) Western blots of SMAD2/3 or control (IgG) immunoprecipitations from nuclear extracts of NANOG KD or control hESCs. Input is 5% of the material used for IP. (F) ChIP-qPCR for the indicated proteins in cells transiently transfected for 3 days with DPY30 shRNA or control shRNA. The results are representative of three independent experiments. The dotted line shows the level of enrichment in the control IgG ChIP (no enrichment). (G) Schematic representation of the location of ChIP-qPCR primers used for histone mark ChIP ("H3K4me3"; also used for H3K4me2, H3K4me1 and H3 ChIP) or transcription factor and histone remodeller ChIP ("S/N/D BS": SMAD2/3, NANOG and DPY30 binding site; also used for WDR5, MLL2/KMT2B, MLL1/KMT2A, SETD1A and BPTF ChIP) relative to the genes to which these regions are associated. Unless otherwise specifically indicated in the figure legends (see Fig. S4E), these ChIP-qPCR primers were used as just described for all figures reported in the study. All ChIP-qPCR primer sequences, the genomic coordinates of the amplicons they generate, and their localization relative to the genes of interest are reported in Table S6.

Bertero_FigS6

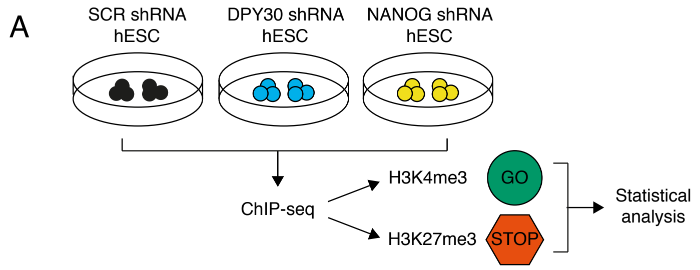

B

Histone mark	Total peaks	Decreased peaks	Increased peaks
**DPY30 shRNA**			
H3K4me3	31923	6482 (20.3%)	1394 (4.4%)
H3K27me3	12728	2669 (17.8%)	2240 (17.6%)
**NANOG shRNA**			
H3K4me3	32642	4028 (12.3%)	1510 (4.6%)
H3K27me3	16152	4580 (28.4%)	3333 (20.6%)

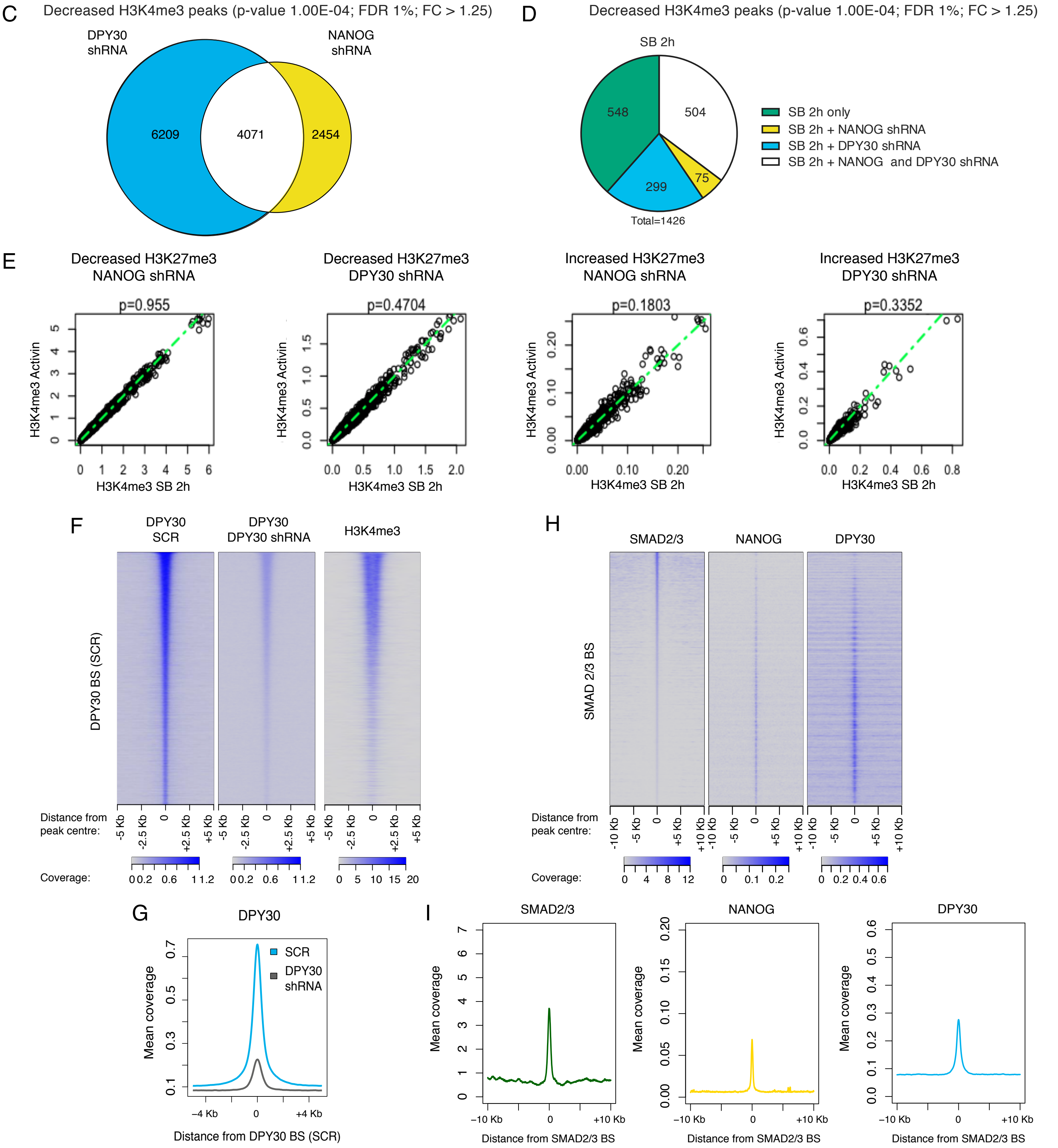

Figure S6, related to Figure 6 and Table S5. SMAD2/3, NANOG and DPY30 control H3K4me3 on a subset of shared genes. (A) Schematics of the experimental approach used to monitor the epigenetic changes associated to DPY30 KD or NANOG KD. (B) Results of the statistical analysis of ChIP-seq data. (C) Overlap between H3K4me3 peaks significantly downregulated after DPY30 KD or NANOG KD. Compared to Fig. 6A, the statistical analysis was performed using less stringent thresholds, as indicated. (D) Proportion of H3K4me3 peaks significantly downregulated after 2h of Activin/Nodal inhibition with SB that were also similarly affected by DPY30 KD, NANOG KD, or both treatments. Compared to Fig. 6B, the statistical analysis was performed using less stringent thresholds, as indicated. (E) Average normalized H3K27me3 read enrichment in three biological replicates before and after inhibition of Activin with SB for 2h. Data refers to H3K27me3 peaks significantly downregulated or upregulated by more than 50% (Benjamini-Hochberg FDR$\leq$0.001) after NANOG KD or DPY30 KD. The level of significant change vs Activin as calculated using Welch's t-test is reported. (F) Heatmaps of coverage for DPY30 ChIP-seq in control hESCs (DPY30, SCR) and DPY30 KD hESCs (DPY30, DPY30 shRNA), and of H3K4me3 in control hESCs (H3K4me3). Coverage was calculated relative to the centre of DPY30 peaks in control hESCs (Table S5). (G) Mean ChIP-seq coverage plot for DPY30 in control (SCR) and DPY30 KD hESCs (DPY30 shRNA). Coverage was calculated relative to the centre of DPY30 peaks in control hESCs. (H) Heatmaps of coverage for SMAD2/3, NANOG and DPY30 ChIP-seq relative to all SMAD2/3 peaks (14085 peaks, Brown et al. 2011). (I) Mean ChIP-seq coverage for all peaks represented in H.

Bertero_FigS7

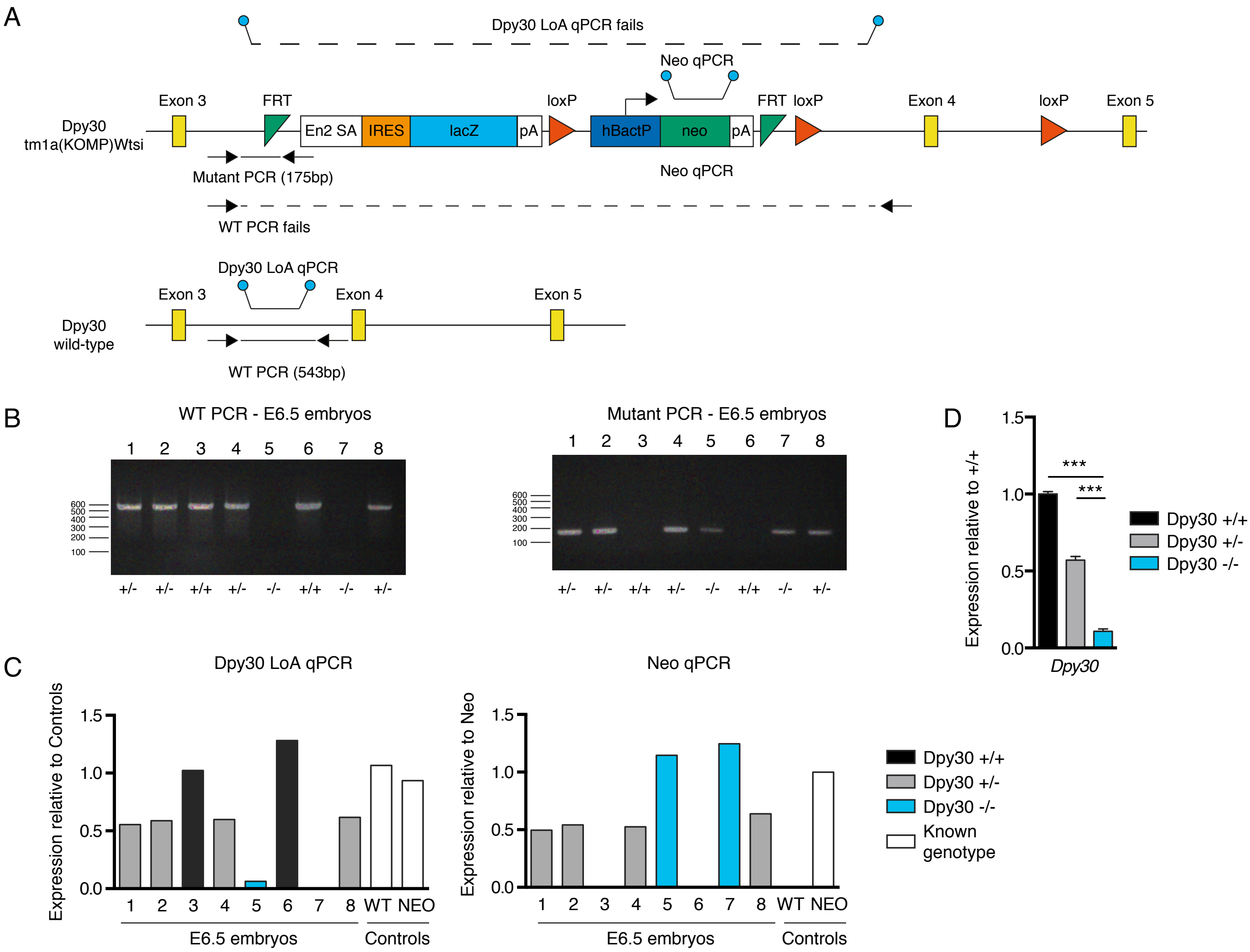

Figure S7, related to Figure 7. Dpy30 is required for mouse post-implantation embryonic development. (A) Schematics of the *Dpy30* “knockout first” allele (Dpy30<tm1a(KOMP)Wtsi) and the wild-type *Dpy30* locus. The location of PCR primers used for genotyping is shown: arrows depict standard PCR, while ball-and-sticks indicate hydrolysis probes-based qPCR. FRT=flippase recognition target site; En2SA=engrailed-2 exon-2 splice acceptor; IRES=internal ribosome entry site; lacZ=β-galactosidase cDNA; pA=poly adenylation site; loxP=bacteriophage P1 Cre recombinase recognition site; hBactP=human β-actin promoter; neo=aminoglycoside phosphotransferase cDNA (neomycin resistance). (B) Representative PCR genotyping of a litter of E6.5 embryos from a Dpy30 mutant heterozygous cross. Molecular weights from a DNA reference

ladder are shown, and the inferred genotypes are reported (+/-=heterozygous; +/+=homozygous wild-type; -/-=homozygous Dpy30 mutant). (C) Representative qPCR genotyping on the same embryos as in B. (D) qPCR for Dpy30 in E6.5 embryos from Dpy30 mutant heterozygous crosses. Significant changes vs Dpy30 +/+ as calculated by one-way ANOVA are reported; n=6 for Dpy30 +/+ and Dpy30 -/-; n=11 for Dpy30 +/-.

SUPPLEMENTAL MATERIAL AND METHODS

hESCs culture.

The H9 hESCs line (WiCell, Madison, WI) was cultured in feeder-free chemically defined conditions on gelatine coated plates as described in (Vallier 2011) with minor modifications described below. The formulation of all cell culture media is detailed in Table S6.

Pluripotent cells were maintained in Chemically Defined Media with BSA (CDM-BSA) supplemented with 10ng/ml recombinant human Activin A and 12ng/ml recombinant human FGF2 (both from Dr. Marko Hyvonen, Dept. of Biochemistry, University of Cambridge). Cells were passaged every 4-6 days with collagenase IV as clumps of 50-100 cells, and dispensed at a density of 100-150 clumps/cm^2. The culture media was replaced 48 hours after the split and then every 24 hours. All assays on undifferentiated hESCs were performed the third day after the split unless otherwise indicated.

In vitro differentiation of hESCs.

Differentiation into endoderm and neuroectoderm was performed as described in (Pauklin and Vallier 2013; Vallier et al. 2009) and initiated in hESCs colonies after 48 hours from the last split. Briefly, neuroectoderm differentiation was performed in CDM-BSA supplemented with 12ng/ml FGF2 and 10μM SB-431542 (Tocris Bioscience), while endoderm specification was obtained in CDM with polyvynilic acid (CDM-PVA) prepared without insulin and supplemented with 50ng/ml FGF2, 1μM Ly-294002 (Promega), 100ng/ml Activin A, and 10ng/ml BMP4 (R&D). Endoderm differentiation was induced for 3 days, while neuroectoderm specification required 6 days.

Pancreatic and hepatic specification was initiated after endoderm differentiation as described in (Cho et al. 2012; Hannan et al. 2013), and details on the differentiation protocols can be found in Table S6. All assays on differentiated hESCs were performed at the end of the differentiation protocol unless otherwise described.

Teratoma assays.

Animal procedures were performed in accordance with the local committee on Animal Experimentation at Centro de Investigación Príncipe Felipe. One million hESCs were injected in the lumen of the right testicle of 6 to 8-weeks-old SCID mice, and three animals were injected in each group. After 12 weeks, mice were sacrificed, and the testicles and tumours were dissected and fixed for 48h in Bouins solution (Sigma-Aldrich). The fixed tissues were then paraffin-embedded and processed according to standard procedures. Sections (5μm) were stained with hematoxylin/eosin and subsequently examined under bright-field microscope for the presence of tissues deriving from the three germ layers.

Genetic manipulation of hESCs.

Knock-down of DPY30 in hESCs was performed as described in (Vallier et al. 2004) by using pLKO.1 vectors expressing specific and validated shRNA (Sigma-Aldrich; clone numbers TRCN0000129317 and TRCN0000131112 for DPY30-sh1 and DPY30-sh2 respectively). A pLKO.1 vector containing a scramble shRNA that does not target any known human gene was used as a control (Sigma-Aldrich; catalogue number SHC016). hESCs were seeded in 6-well plates and transfected 48 hours after the split with 4μg of pLKO.1 using Lipofectamine 2000 (Invitrogen) for 24 hours following manufacturer's instructions.

For stable transfections, 1mg/ml Puromycin (Gibco) was added to the media from 5 days after the transfection onwards to allow for selection of stable integrants. After 10 days of selection, colonies derived from a single event of stable integration were counted and morphologically assessed for pluripotency or differentiation. Single colonies were then picked and clonally expanded. The level of knock-down was determined by qPCR and confirmed by Western blot as described below. Stable NANOG knock-down hESCs and matched controls were previously obtained using this same protocol as described in (Teo et al. 2011).

In the case of transient transfections, selection with Puromycin was initiated immediately after transfection and maintained for one, two or three days.

Alkaline phosphatase assay.

hESCs were fixed with 4% PFA in PBS (Alfa Aesar) for 20 minutes at 4°C, washed three times with PBS, and rinsed with Alkaline Phosphatase (AP) solution (100mM Tris-HCl ph9.5, 100mM NaCl). The AP reaction was performed by incubating cells for 20 minutes at room temperature with AP solution supplemented with Nitro Blue Tetrazolium (NBT) and 5-bromo-4-chloro-3indolyl-phospatase (BCIP) at a ratio of 33µl of NBT and 16µl BCIP per 5ml AP solution (NBT/BCIP colour development substrate, Promega). After staining, cells were washed three times with PBS and imaged using an Axiovert 200M inverted microscope (Zeiss).

Proliferation curve.

hESCs were plated in four biological replicates for each time point and fixed in PBS 4% PFA for 20 minutes at 4°C at intervals of 24 hours for four days starting from the day after the split (D0). After being washed three times with PBS, cells were stained for 10 minutes at room temperature with a solution of 0.1% Crystal Violet (Sigma) in deionized water. Plates were then washed three times with deionized water for 10 minutes at room temperature, air-dried and stored protected from light. The dye was dissolved in 0.5ml of 10% Acetic Acid (Sigma), and the absorbance at 590nm was measured using an Envision Multilabel Reader (Perkin Elmer). The data, which is proportional to the number of cells, was normalized to the average absorbance at D0.

Apoptosis assay.

Apoptosis was measured 12 hours after the last media change by using the ApoTox-Glo Triplex Assay from Promega according to manufacturer's instructions. Briefly, cells were first incubated for 30 minutes at 37°C with the Viability/Cytotoxicity reagent containing the GF-AFC and bis-AAF-R110 substrate and fluorescence was measured at $400_{ex}/505_{em}$ (Viability) and $485_{ex}/520_{em}$ (Citotoxicity) using an Envision Multilabel Reader. Then, cells were further incubated for 30 minutes at room temperature with the Caspase-Glo 3/7 Reagent, and Caspase 9 activation was measured as luminescence using a Glomax 96-Microplate Luminometer.

Measurements were performed on four biological replicates and expressed as average Caspase activation/Viability for each condition.

Gene expression analysis by quantitative real-time PCR (qPCR).

Cellular RNA was extracted using the GenElute Mammalian Total RNA Miniprep Kit (Sigma-Aldrich) and contaminating genomic DNA was removed using the On-Column DNase I Digestion Set (Sigma-Aldrich) following manufacturer's instructions. 500ng of RNA was used for cDNA synthesis in a reaction containing 250ng random primers, 0.5mM dNTPs, 20U RNaseOUT and 25U of SuperScript II (all from Invitrogen) in a total of 20µl according to manufacturer's instructions.

cDNA was diluted 30 fold and 5µl were used for qPCR using SensiMix SYBR low-ROX (Bioline) and 150nM forward and reverse primers (Sigma-Aldrich; see Table S6 for all primer sequences). Samples from three biological replicates for each condition were run in technical duplicates on 96-well plates on a Stratagene Mx-3005P (Agilent) and results were analysed using the ΔΔCt approach using *HMBS/PBGD* as housekeeping gene (Livak and Schmittgen 2001). The reference sample used as control to calculate gene expression is indicated in each figure. In cases where multiple control samples were used for one experiment, the average ΔCt from all controls was used during this calculation. Data was expressed as average relative gene expression ± SEM. All primers were designed using PrimerBlast (http://www.ncbi.nlm.nih.gov/tools/primer-blast/) and validated to have a PCR efficiency >98% as well as to produce a single PCR product.

Protein expression analysis by Western blot.

In order to prepare total protein lysates, cells were harvested by scraping in PBS, centrifuged at 250g for 5 minutes at 4°C, and re-suspended in ice-cold CellLytic M buffer (Sigma) supplemented with cOmplete Protease Inhibitor (Roche). After an incubation for 30 minutes at 4°C on a rotating wheel, lysates were clarified by centrifugation at 16000g for 10 minutes at 4°C. The supernatant was transferred to a new tube, and protein concentration was assessed using Protein Quantification Kit-Rapid (Sigma).

Samples were prepared for Western Blot analysis by adding Laemly Buffer (final concentration: 30mM Tris-HCl pH 6.8, 6% Glycerol, 2% SDS, 0.02% Bromophenol Blue, 0.25% β-mercaptoethanol), and denatured at 95°C for 5 minutes. 20µg of proteins were loaded and run on 4-12% NuPAGE Bis-Tris Precasts Gels (Invitrogen), then transferred to PVDF membranes by liquid transfer using NuPAGE Transfer Buffer (Invitrogen). Membranes were blocked for 1 hour at room temperature in PBS 0.05% Tween (PBST) supplemented with 4% non-fat dried milk, and incubated overnight at 4°C with the primary antibody diluted in the same blocking buffer (Table S6 contains details on all primary and secondary antibodies used for Western Blot and other applications). After three washes in PBST, membranes were incubated for 1 hour at room temperature with HRP-conjugated secondary antibodies diluted in blocking buffer, then further washed three times with PBST before being incubated with Pierce ECL2 Western Blotting Substrate (Thermo) and exposed to X-Ray Super RX Films (Fujifilm).

Immunostaining.

Cells were fixed for 20 minutes at 4°C in PBS 4% PFA, rinsed three times with PBS, and blocked and permeabilized at the same time for 30 minutes at room temperature using PBS with 10% Donkey Serum (Biorad) and 0.1% Triton X-100 (Sigma). Overnight incubation at 4°C with the primary antibodies (Table S6) diluted in PBS 1% Donkey Serum 0.1% Triton X-100 was followed by three washes with PBS, and by further incubation with AlexaFluor secondary antibodies (Table S6) for 1 hour at room temperature protected from light. Cells were finally washed three times with PBS, and DAPI (Sigma) was added to the first wash to stain nuclei. Images were acquired using a LSM 700 confocal microscope (Leica).

Flow-cytometry.

Single cell suspensions were prepared by incubation in Cell Dissociation Buffer (Gibco) for 10 minutes at 37° followed by extensive pipetting. Cells were washed twice with PBS and fixed for 20 minutes at 4°C with PBS 4% PFA. After three washes with PBS, cells were first permeabilized for 20 minutes at room temperature

with PBS 0.1% Triton X-100, then blocked for 30 minutes at room temperature with PBS 10% Donkey Serum. Primary and secondary antibodies incubations (Table S6) were performed for 1 hour each at room temperature in PBS 1% Donkey Serum 0.1% Triton X-100, and cells were washed three times with this same buffer after each incubation. Flow-cytometry was performed using a Cyan ADP flow-cytometer and at least 10.000 events were recorded.

Immunoprecipitation (IP).

All steps were performed on ice or at 4°C and ice-cold buffers (compositions detailed in Table S6) supplemented with cOmplete Protease Inhibitors (Roche), PhosSTOP Phosphatase Inhibitor Cocktail (Roche), 1mg/ml Leupeptin, 0.2mM DTT, 0.2mM PMSF, and 10mM NaButyrate (all from Sigma) were used unless otherwise stated. Cells were fed with fresh media for 2 hours before being washed with PBS, scraped in Cell Dissociation Buffer and pelleted at 250g for 10 minutes. The cell pellet was incubated in five volumes of ILB for 10 minutes to allow for cells to swell, then Triton X-100 was added to a final concentration of 0.3% and cells were incubated for 6 further minutes to lyse the plasma membrane. Nuclei were pelleted at 600g for 5 minutes, washed once with ten volumes of ILB, and finally re-suspended in two volumes of NLB. The nuclear suspension was transferred to a Dounce Homogenizer (Jencons Scientific) and homogenized by performing 70 strokes with a "tight" pestle. The nuclear lysate was first incubated in rotation for 30 minutes, then 125U/ml of Benzonase Nuclease (Sigma) were added to digest nucleic acids for 45 at room temperature. The lysate was clarified by ultracentrifugation at 180.000g for 30 minutes and pre-cleared by incubation with non-immune IgG (R&D) for one hour in rotation followed by two extra hours with Protein G-Agarose (Roche). The protein concentration was assessed, and 1mg of protein was used for overnight IP with the primary antibody in rotation (Table S6). IP were further incubated for one hour with 15μl of Protein G-Agarose, washed five times with NLB, and resuspended in Laemly Buffer. Samples were analysed by Western Blot as described above.

Chromatin immunoprecipitation (ChIP).

All steps were performed on ice or at 4°C and ice-cold buffers (compositions detailed in Table S6) supplemented with 1mg/ml Leupeptin, 0.2mM PMSF, and 10mM NaButyrate were used unless otherwise stated. Cells were fed with fresh media for 2 hours before the beginning of the experiment. $2x10^7$ cells were used for transcription factors ChIP (ChIP-XL), and $1.5x10^6$ cells were used for histone marks ChIP. Cells for ChIP-XL were cross-linked on plates first with protein crosslinkers (10mM dimethyl 3,3'-dithiopropionimidate dihydrochloride and 2.5mM 3,3'-dithiodipropionic acid di-N-hydroxysuccinimide ester; Sigma) for 15 minutes at room temperature, then with 1% formaldehyde for 15 more minutes, as described in (Brown et al. 2011). Cells for histone ChIP were cross-linked only with 1% formaldehyde for 15 minutes. Cross-linking was stopped by adding glycine to a final concentration of 0.125M followed by incubation for 10 minutes at room temperature, and the cells were washed with PBS before being scraped off the plates in PBS. The cell suspension was centrifuged at 250g for 5 minutes, and the pellet was re-suspended and incubated for 10 minutes in 2ml of CLB to lyse the plasma membranes. Nuclei were pelleted at 600g for 5minutes and lysed in 1.25ml of SNLB for 10 minutes, after which 0.75ml of IDB were added. Chromatin was sonicated using a S-400 Ultrasonic Liquid Processor (Misonix) equipped with a 1-16" dia Ultrasonic Cell Disruptor Microtip Probe (Cole-Palmer) by doing cycles of 15 seconds ON, 45 seconds OFF at 60% amplitude; 20 and 12 cycles were performed for ChIP-XL and standard ChIP respectively. This protocol resulted in the homogeneous generation of fragments of 100-400bp. Samples were clarified by centrifugation at 16000g for 10 minutes, and diluted with 3.5ml of IDB. After pre-clearing with 25µg of non-immune IgG for 1h and 50µl of Protein G-Agarose for 2h, ChIP was performed overnight in rotation using specific antibodies (Table S6) or non-immune IgG as a control. After incubation for 1 hour with 30µl of Protein G-Agarose, beads were washed twice with IWB1, once with IWB2 and twice with TE. Precipitated DNA was eluted with 150µl of EB twice for 15 minutes at room temperature in rotation, and processed as follows in parallel with 300µl of sonicated chromatin non-used for ChIP (input). Protein-protein cross-linking in ChIP-XL samples was first reverted by adding DTT to a concentration of 100µM and incubating for 30 minutes at 37°C, then samples were incubated at 65°C for 5

hours after adding NaCl to a final concentration of 300mM for protein-DNA de-crosslinking and 1μg RNase A (Sigma) to digest contaminating RNA. Finally, 60μg of Proteinase K (Sigma) were added overnight at 45°C. DNA was extracted by sequential phenol-chloroform and chloroform extractions using Phase Lock Gel Heavy tubes (5Prime), and precipitated overnight at -80°C in 100mM NaAcetate and 66% ethanol; 50μg of glycogen (Ambion) were added as carrier. After centrifugation at 16.000g for 1 hour at 4°C, DNA pellets were washed once with ice-cold 70% ethanol, and finally air-dried. ChIP and inputs were resuspended in 300μl and 700μl respectively and 5μl were used for qPCR as described above. Results of transcription factor ChIP were calculated using the ΔΔCt approach using a region in the last exon of *SMAD7* to normalize for background binding (Table S6), and normalizing the enrichment to the one of non-immune IgG ChIP. Histone ChIP data were expressed as % of input DNA by calculating (Ct_{chip}-Ct_{input})^2 and correcting for the dilutions performed. On top of this, for some graphs histone ChIP enrichments were normalized to a control condition to facilitate visualization and interpretation of the results.

Sequential ChIP

Sequential ChIP was performed as described in (Truax and Greer 2012) with minor modifications. Briefly, a first round of ChIP-XL was performed as described above, but elution of the immunoprecipitated material was done for 30' at 37 °C using 75ul of TE buffer supplemented with 1% SDS, fresh 15mM DTT and cOmplete Protease Inhibitors (Roche). The beads were then washed once with 75ul of IDB and this second eluate was pooled with the first. The total eluate (150ul) was diluted 10 times in IDB, incubated at 4 °C for five hours, and then subjected to a second round of ChIP as described above. Purified sequential ChIP material was resuspended in 100μl and 5μl were used for qPCR as described above, with the exception that KAPA SYBR FAST qPCR Master Mix (KAPA Biosystems) was used, as this proved to provide higher sensitivity and reliability in detecting lower amounts of DNA obtained from sequential ChIP. Data was analyzed as described above, and the enrichments were normalized to those obtained for a control sequential ChIP where the first ChIP using the antibody of interest (anti-DPY30) was followed by a second round of ChIP using non-immune IgG.

Microarrays.

Cellular RNA was extracted as described above, and 500ng were amplified and purified using the Illumina TotalPrep-96 RNA Amplification kit (Life Technologies) according to the manufacturer's instructions. Three biological replicates for each condition were analysed. Biotin-Labelled cRNA was then normalized to a concentration of 150ng/µl and 750ng were hybridised to Illumina Human-12 v4 BeadChips for 16 hours (overnight) at 58 °C. Following hybridisation, BeadChips were washed and stained with streptavidin-Cy3 (GE Healthcare). BeadChips were then scanned using the BeadArray reader, and image data was then processed using Genome Studio software (Illumina). The raw and processed microarray data are available on ArrayExpress (Accession number: E-MTAB-2749).

Microarrays analysis.

Probe summaries for all arrays were obtained from the raw data using the method "Making Probe Summary". These values were transformed (variance stabilized) and quantile normalised using the R/Bioconductor package lumi (Du et al. 2008). Standard lumi QC procedure was applied and no outliers were identified.

The analysis of differentially expressed probes during the time-course of Activin inhibition was performed using the R/Bioconductor timecourse package (Smyth 2004). The Hotelling T^2 score for each probe that fluoresced above background in at least one condition was calculated using the mb.long function with all parameters set to their default value. We used the T^2 score to rank probes according to differential expression across the time-course. The top 10% probes were selected for complete Eucledian hierarchical clustering (k-means preprocessing; max of 300 clusters) using Perseus software (MaxQuant); Z-scores of the $\log_2$ normalized expression values across the time-course were calculated and used for this analysis. 19 probe clusters (having an Euclidean distance >3.68) were defined, and gene enrichment analysis of genes in selected clusters was performed using Enrichr (Chen et al. 2013). The significance of the overlap between genes associated to

SMAD2/3 binding sites (Brown et al. 2011) and genes in selected clusters was measured with hypergeometric tests considering the sampling population as the number of genes whose expression was measured by the microarray.

Differential expression between pairs of conditions was evaluated using the R/Bioconductor package limma (Smyth 2004). A linear model fit was applied, and the top differentially expressed genes were tabulated for each contrast using the method of Benjamini and Hochberg to correct the p-values (Benjamini and Hochberg 1995). Probes that failed to fluoresce above background in both conditions were removed. Differentially expressed probes were selected using a cutoff of adjusted p-value <0.05 and absolute fold-change > 1.35 (the recommended threshold for confident measurement of differential expression according to the microarray chip manufacturer).

Rank-rank hypergeometric overlap analysis (RRHO, Plaisier et al., 2010) was used to measure the significance of the overlap between genes up- or downregulated in pairs of treatment-control experiments. All probes that significantly fluoresced above background in at least one condition were ranked by their $\log_2$ fold-change of normalized expression in each experiment. This raking was used as input for the online version of RRHO to calculate Benjamini-corrected hypergeometric probabilities (step size = 150). Heatmaps reported in Fig. 3G and Fig. S5B were obtained by plotting Z-scores of the $\log_2$ normalized expression values calculated within each experiments separately (an experiment including triplicate samples for a treatment and a control condition). The data was subjected to Eucledian hierarchical clustering as described above, but only for rows.

ChIP sequencing.

For histone mark ChIP-sequencing, ChIP was performed as described above on three biological replicates per condition analyzed; 7.5µg of fragmented chromatin (corresponding to roughly 1.5×10^6 hESCs) were used per ChIP with anti-H3K4me3 or anti-H3K27me3 antibodies (Table S6). For DPY30 ChIP-sequencing, ChIP-XL was performed as described above on one biological replicate per condition analyzed; 2×10^7 hESCs were used with 5µg of anti-DPY30 antibody (Table S6). At the end of the ChIP protocol, fragments between 100bp

and 400bp (average length 200bp) were used to prepare barcoded sequencing libraries using NEBNext Sample Prep Kit1 (New England BioLabs) following manufacturer's instructions. 10ng of input material for each condition were also used for library preparation and later used as a control during peak calls. Equimolar amounts of each library were pooled and this multiplexed library was diluted to 8pM before sequencing using an Illumina HiSeq 2000 performing 75bp paired-end reads. The raw and processed ChIP-seq data are publicly available on ArrayExpress (Accession number: E-ERAD-191: histone mark ChIP-sequencing; E-ERAD-365: DPY30 ChIP-sequencing).

ChIP-seq data analysis.

Reads were mapped to 1000 Genomes Phase II (hs37d5) reference assembly using BWA (Li and Durbin 2009). Only reads mapped to chromosomes 1-22, X and Y with MAPQ values higher or equal than 20 were kept for further processing.

Peak calling for H3K4me3 ChIP-seq was done versus input controls using PeakRanger 1.16 (Feng et al. 2011). In order to follow the recommendation of control samples having substantially deeper sequencing depth than treatment samples in chromatin modification ChIP-seq (Bailey et al. 2013), we pooled the input control samples. No substantial differences were found when repeating the analysis without pooling the control samples (≈99% peaks shared with respect to the pooled control). Reads were extended to 250 bp and smoothing bandwidth increased to 200 to accommodate sharp histone marks instead of default mode for narrow peaks produced by transcription factors (-l 250 -b 200 -q 0.05). Chromosomal locations of significant read-enrichment at FDR ≤ 0.05 were kept for further analysis. Peak calling for DPY30 ChIP-seq was done also with PeakRanger (-l 250 -b 150 -q 0.05 -p 0.0000001) using as a control a combined file of INPUT (scramble shRNA) and INPUT KD (DPY30 shRNA). Peak calling for H3K27me3 was done using the CCAT algorithm (Xu et al., 2010) included in the PeakRanger software suite with a sliding window of 1000 bp and FDR ≤ 0.05 (-win_size 1000 --win_step 100 --min_count 70 --min_score 7 -l 250 -q 0.05).

For visualization purposes, biological replicates of histone mark ChIP-seq were combined. Normalized

bedGraph format files were produced for each sample using BEDTools 2.17.0 (Quinlan and Hall 2010). The reads mapped at both DNA strands from 5' to 3' direction were extended to a length of 250 bp, and the read count at each genomic position was normalized to the library size and per million reads (multiplying every value by '1,000,000 / number_of_mapped_reads'). bedGraph files were converted to bigWig using UCSC tool bedGraphToBigWig, and visualized on the Biodalliance genome viewer (Down et al. 2011) to generate figures.

To assess the reproducibility of the histone mark ChIP-seq peaks, we computed the Pearson correlation coefficient (PCC) between H3K4me3 and H3K27me3 pairs of sequenced samples using the tool bigWigCorrelate (UCSC) over an aggregated list of peaks at each histone mark. All replicates used for further analysis have PCC > 0.98 for H3K4me3, and PCC > 0.95 for H3K27me3, respectively. Three replicates were used for each condition except for H3K27me3 ChIP-seq in NANOG KD and its matched Scramble control, where only two replicates were considered because the third showed lower levels of reproducibility (PCC<0.74). We computed the PCC values for the complete genome at a base-pair resolution and obtained similar results. Finally, only peaks regions reproducible at biological replicates passing PCC analysis were kept. Those located equal to or closer than 500 bp (H3K4me3) or 5 Kb (H3K27me3) were merged.

ChIP-seq peaks were annotated using the R/Bioconductor package ChIPpeakAnno (Zhu et al. 2010) using the dataset "hsapiens_gene_ensembl" from ensembl. Proximal promoter and immediate downstream were considered respectively 1Kb upstream or downstream the TSS. GREAT analysis was performed as previously described (McLean et al. 2010) using all parameters as standard.

Negative Binomial tests implemented in diffReps (Shen et al. 2013) were used to detect differential histone modification regions using a sliding window of 600bp (H3K4me3) or 5000bp (H3K27me3), p-value cutoff 1e-6, sharp peaks mode for H3K4me3 (--nsb 20) and broad peak mode for H3K27me3 (--nsb 2), hg19 as reference genome, and an average fragment size of 250bp (rest of parameters default). Differential histone modifications regions not overlapping (at least 1bp) significant chromatin marks previously detected during peak calling at least in one of conditions under comparison were removed. Regions were ranked by their fold-change (FC), and reported as differentially enriched only if the absolute FC≥1.5, and Benjamini-Hochberg corrected p-value≤1e-

3. As described on the figures, figure legends and results, for Fig. S6C and S6D only the thresholds for statistical significance were relaxed as it follows: binomial test p-value cutoff 1e-4 (diffReps standard); absolute FC≥1.25, and Benjamini-Hochberg corrected p-value≤1e-2 (FDR 1%).

Integration of ChIP-seq datasets.

The significance of the overlap between H3K4me3 peaks decreased after 2h of SB treatment and SMAD2/3 binding sites was calculated using GAT (Heger et al. 2013), with the annotations being SMAD2/3 binding sites (Brown et al. 2011), the segments being H3K4me3 regions, and the workspace being the hg19 genome after subtraction of the hg19/GRCh37 ENCODE Excludable Mappability Regions (Bernstein et al., 2012; number of samples to compute = 10k). To enable the comparison, SMAD2/3 binding sites coordinates were translated from hg18 to hg19 using UCSC liftOver under default parameters.

The overlap of different histone marks was evaluated using the function intersect (with parameter –u) from the BEDTools suite (Quinlan and Hall 2010). Bed format files for H3K4me3 and H3K27me3 peaks were obtained from the reproducible peak calls described above, while data for H3K4me1 and H3K27me3 in H1 hESCs from the ENCODE project was downloaded from the UCSC browser.

The significance of the multiple overlaps between H3K4me3 peaks decreased after 2h of SB treatment, DPY30 KD or NANOG KD was calculated using MULTOVL (Aszódi 2012). We pooled the total set of H3K4me3 regions for all conditions considered as reported by the diffReps analysis described above, and considered them as the free space in the tool multovlprob included in MULTOVL. We randomly reshuffled 10,000 times down-regulated regions in H3K4me3 to estimate a null distribution of overlap length. P-values were estimated from the Z-scores as described in (Aszódi 2012).

Quantitative changes of H3K4me3 and H3K27me3 levels on selected genomic regions were obtained by computing for each region a score from the normalized coverage as follows: for each region $i=1,\ldots,L$, where L is total number of regions considered, we obtained the score S_i from the normalized coverage (bigwig data, as described above) $y_{i,j} \in [x_{i,a}, x_{i,b}]$ for a replicate $j=1,2,3$ as

$$S_{i,j} = \frac{\sum_{w=x_{i,a}}^{x_{i,b}} y_{i,j}(w)}{x_{i.b} - x_{i,a}}$$

where $x_{i,a}$ is the start location of the peak region, and $x_{i,b}$ the end. Scores were calculated for ChIP-seq in the presence of Activin or after 2h of SB treatment, and significant differences in their mean values were assessed by Welch's t-test for two-samples. This analysis was performed on different sets of genomic regions described in the text and figure legends.

Average meta-region plots and coverage heatmaps for NANOG, SMAD2/3 and DPY30 ChIP-seq were done using the genomation toolkit (Akalin et al. 2014). 1000 bins were used for each window, and a winsorize configuration of (0,99) to limit the values to only 99th percentile for a matrix (everything above the 99 percentile was equalized to the value of 99th percentile). Signal track for NANOG in H1 hESCs was obtained from the ENCODE project (Bernstein et al. 2012). Genome-wide coverage for SMAD2/3 was calculated by combining the alignments in BED format in GEO Series GSE19461 (Brown et al. 2011), using the function 'genomecov' in the BEDTools suite (Quinlan and Hall 2010), and then converting to bigwig format. 'bwtool lift' (Pohl and Beato 2014) was used to lift hg18 to hg19 coordinates to allow inter-dataset comparison, using the liftOver chain file hg18ToHg19.over.chain.gz downloaded from UCSC. The subset of SMAD2/3 binding sites in Fig 6H and 6I was obtained overlapping SMAD2/3 peaks with regions obtained extending +/- 100 Kb H3K4me3 peaks downregulated after SB treatment.

Integration of ChIP-seq and microarray data.

Each H3K4me3 peak was mapped to its closest gene using the annotatePeaks.pl function from the HOMER suite (Heinz et al. 2010) with standard parameters, and using the UCSC hg19 annotation. The 491 H3K4me3 peaks downregulated after 2h of SB mapped to 415 unique genes. The difference in expression of microarray probes mapping to genes associated to differentially enriched H3K4me3 was statistically evaluated by performing Welch's paired two-samples t-tests. In the case of the SB treatment time-course Friedman's non-

parametric paired test was performed to evaluate the significance of overall changes, and Dunn's corrected multiple comparisons were performed for selected pairs of conditions.

GSEA (Gene Set Enrichment Analysis; Subramanian et al., 2005) was performed by generating a custom gene set file containing genes significantly downregulated after 48h of SB treatment (adj.p<0.05 and fold-change<-1.35, calculated with limma package as described above), and testing the enrichment of this gene set in the list of genes closest to H3K4me3 peaks ranked by the $\log_2$ fold-change of the enrichment in the treatment vs control (in cases where multiple H3K4me3 peaks mapped to the same gene, the average $\log_2$ fold-change was considered).

Generation of knockout mice.

All animal procedures were performed in agreement with the UK Home Office regulations, UK Animals (Scientific Procedures) Act of 1996. Mice carrying the *Dpy30* "knockout first" tm1a(KOMP)Wtsi allele were generated by the Sanger Institute Mouse Genetics Project as described previously (Skarnes et al. 2011; White et al. 2013). Briefly, mouse C57BL/6N ES cells were electroporated with a linearized "knockout-first" plasmid vector carrying a IRES:LacZ trapping cassette and a floxed promoter-driven neo cassette inserted between two homology arms mapping to exons 3 and 4 of *Dpy30*. Cells were selected for integration using Geneticin (Invitrogen), expanded and screened with a panel of tests to confirm site-specific homologous recombination (loxP site confirmation qPCR; lacZ confirmation qPCR; loss of wild-type allele copy number qPCR; Valenzuela et al., 2003).

After screening, targeted mouse ESC were microinjected in C57BL/6 blastocyst to obtain chimeras. Germline transmission was detected by neo copy number qPCR and additional quality control tests performed to confirm the structure and targeting of the allele (Ryder et al. 2013). Heterozygotes were crossed with C57BL/6NTac mice and the colony was expanded before intercrossing.

Phenotyping of knockout mice.

The viability of homozygous Dpy30 knockout mice was tested by crossing heterozygous animals and genotyping the offspring at postnatal day 14 and embryonic days encompassing 6.5 to 14.5. Genotype was confirmed both by *neo* copy number qPCR and loss of wild-type allele copy number qPCR (Table S6) on DNA extracted from earclips using the TaqMan Sample-to-SNP kit (Life Technologies). Embryos were dissected from the decidua of pregnant females and all extraembryonic tissues were removed. Images were acquired using a Leica MZ16A microscope.

In the case of E6.5 and E7.5 embryos, DNA and RNA were simultaneously extracted from whole embryos using the AllPrep DNA/RNA Mini Kit (Qiagen) according to manufacturers instructions. DNA was eluted in 25μl and used for both qPCR as described above and standard PCR to confirm the presence of wild-type and mutant alleles. RNA was eluted in 15μl and used for cDNA synteshis and then qPCR to measure gene expression as described above. Gene expression was measured with the ΔΔCt approach using the geometric mean of *HMBS/PBGD* and *GAPDH* Ct values as housekeeping reference.

SUPPLEMENTAL REFERENCES

Akalin A, Franke V, Vlahoviček K, Mason CE, Schübeler D. 2014. Genomation: a Toolkit To Summarize, Annotate and Visualize Genomic Intervals. *Bioinformatics* 1–3.

Aszódi A. 2012. MULTOVL: fast multiple overlaps of genomic regions. *Bioinformatics* **28**: 3318–9.

Bailey T, Krajewski P, Ladunga I, Lefebvre C, Li Q, Liu T, Madrigal P, Taslim C, Zhang J. 2013. Practical Guidelines for the Comprehensive Analysis of ChIP-seq Data ed. F. Lewitter. *PLoS Comput Biol* **9**: e1003326.

Benjamini Y, Hochberg Y. 1995. Controlling the False Discovery Rate : A Practical and Powerful Approach to Multiple Testing. *J R Stat Soc* **57**: 289–300.

Bernstein BE, Birney E, Dunham I, Green ED, Gunter C, Snyder M. 2012. An integrated encyclopedia of DNA elements in the human genome. *Nature* **489**: 57–74.

Brown S, Teo A, Pauklin S, Hannan N, Cho CH-H, Lim B, Vardy L, Dunn NR, Trotter M, Pedersen R, et al. 2011. Activin/Nodal signaling controls divergent transcriptional networks in human embryonic stem cells and in endoderm progenitors. *Stem Cells* **29**: 1176–85.

Chen EY, Tan CM, Kou Y, Duan Q, Wang Z, Meirelles GV, Clark NR, Ma'ayan A. 2013. Enrichr: interactive and collaborative HTML5 gene list enrichment analysis tool. *BMC Bioinformatics* **14**: 128.

Cho CH-H, Hannan NR-F, Docherty FM, Docherty HM, Joåo Lima M, Trotter MWB, Docherty K, Vallier L. 2012. Inhibition of activin/nodal signalling is necessary for pancreatic differentiation of human pluripotent stem cells. *Diabetologia* **55**: 3284–95.

Down TA, Piipari M, Hubbard TJP. 2011. Dalliance: interactive genome viewing on the web. *Bioinformatics* **27**: 889–90.

Du P, Kibbe W a, Lin SM. 2008. lumi: a pipeline for processing Illumina microarray. *Bioinformatics* **24**: 1547–8.

Feng X, Grossman R, Stein L. 2011. PeakRanger: a cloud-enabled peak caller for ChIP-seq data. *BMC Bioinformatics* **12**: 139.

Hannan NRF, Segeritz C-P, Touboul T, Vallier L. 2013. Production of hepatocyte-like cells from human pluripotent stem cells. *Nat Protoc* **8**: 430–7.

Heger A, Webber C, Goodson M, Ponting CP, Lunter G. 2013. GAT: a simulation framework for testing the association of genomic intervals. *Bioinformatics* **29**: 2046–8.

Heinz S, Benner C, Spann N, Bertolino E, Lin YC, Laslo P, Cheng JX, Murre C, Singh H, Glass CK. 2010. Simple combinations of lineage-determining transcription factors prime

cis-regulatory elements required for macrophage and B cell identities. *Mol Cell* **38**: 576–89.

Li H, Durbin R. 2009. Fast and accurate short read alignment with Burrows-Wheeler transform. *Bioinformatics* **25**: 1754–60.

Livak KJ, Schmittgen TD. 2001. Analysis of relative gene expression data using real-time quantitative PCR and the 2(-Delta Delta C(T)) Method. *Methods San Diego Calif* **25**: 402–408.

McLean CY, Bristor D, Hiller M, Clarke SL, Schaar BT, Lowe CB, Wenger AM, Bejerano G. 2010. GREAT improves functional interpretation of cis-regulatory regions. *Nat Biotechnol* **28**: 495–501.

Pauklin S, Vallier L. 2013. The Cell-Cycle State of Stem Cells Determines Cell Fate Propensity. *Cell* **155**: 135–147.

Plaisier SB, Taschereau R, Wong J a, Graeber TG. 2010. Rank-rank hypergeometric overlap: identification of statistically significant overlap between gene-expression signatures. *Nucleic Acids Res* **38**: e169.

Pohl A, Beato M. 2014. bwtool: a tool for bigWig files. *Bioinformatics* **30**: 1618–9.

Quinlan AR, Hall IM. 2010. BEDTools: a flexible suite of utilities for comparing genomic features. *Bioinformatics* **26**: 841–2.

Ryder E, Gleeson D, Sethi D, Vyas S, Miklejewska E, Dalvi P, Habib B, Cook R, Hardy M, Jhaveri K, et al. 2013. Molecular characterization of mutant mouse strains generated from the EUCOMM/KOMP-CSD ES cell resource. *Mamm Genome* **24**: 286–94.

Shen L, Shao N-Y, Liu X, Maze I, Feng J, Nestler EJ. 2013. diffReps: detecting differential chromatin modification sites from ChIP-seq data with biological replicates. *PLoS One* **8**: e65598.

Skarnes WC, Rosen B, West AP, Koutsourakis M, Bushell W, Iyer V, Mujica AO, Thomas M, Harrow J, Cox T, et al. 2011. A conditional knockout resource for the genome-wide study of mouse gene function. *Nature* **474**: 337–42.

Smyth GK. 2004. Linear models and empirical bayes methods for assessing differential expression in microarray experiments. *Stat Appl Genet Mol Biol* **3**.

Subramanian A, Tamayo P, Mootha VK, Mukherjee S, Ebert BL. 2005. Gene set enrichment analysis : A knowledge-based approach for interpreting genome-wide expression profiles. *Proc Natl Acad Sci U S A* **102**: 15545–15550.

Teo A, Arnold SJ, Trotter MWB, Brown S, Ang LT, Chng Z, Robertson EJ, Dunn NR, Vallier L. 2011. Pluripotency factors regulate definitive endoderm specification through eomesodermin. *Genes Dev* **2**: 238–250.

Truax AD, Greer SF. 2012. ChIP and Re-ChIP Assays: Investigating Interactions Between Regulatory Proteins, Histone Modifi cations, and the DNA Sequences to Which They Bind ed. A. Vancura. *Methods Mol Biol* **809**: 175–188.

Valenzuela DM, Murphy AJ, Frendewey D, Gale NW, Economides AN, Auerbach W, Poueymirou WT, Adams NC, Rojas J, Yasenchak J, et al. 2003. High-throughput engineering of the mouse genome coupled with high-resolution expression analysis. *Nat Biotechnol* **21**: 652–9.

Vallier L. 2011. Serum-Free and Feeder-Free Culture Conditions for Human Embryonic Stem Cells ed. N.I. Nieden. *Springer Protoc* **690**: 57–66.

Vallier L, Rugg-Gunn PJ, Bouhon IA, Andersson FK, Sadler AJ, Pedersen RA. 2004. Enhancing and diminishing gene function in human embryonic stem cells. *Stem Cells* **22**: 2–11.

Vallier L, Touboul T, Brown S, Cho C, Bilican B, Alexander M, Cedervall J, Chandran S, Ahrlund-Richter L, Weber A, et al. 2009. Signaling pathways controlling pluripotency and early cell fate decisions of human induced pluripotent stem cells. *Stem Cells* **27**: 2655–66.

White JK, Gerdin A-K, Karp N a, Ryder E, Buljan M, Bussell JN, Salisbury J, Clare S, Ingham NJ, Podrini C, et al. 2013. Genome-wide generation and systematic phenotyping of knockout mice reveals new roles for many genes. *Cell* **154**: 452–64.

Xu H, Handoko L, Wei X, Ye C, Sheng J, Wei C-L, Lin F, Sung W-K. 2010. A signal-noise model for significance analysis of ChIP-seq with negative control. *Bioinformatics* **26**: 1199–204.

Zhu LJ, Gazin C, Lawson ND, Pagès H, Lin SM, Lapointe DS, Green MR. 2010. ChIPpeakAnno: a Bioconductor package to annotate ChIP-seq and ChIP-chip data. *BMC Bioinformatics* **11**: 237.
